# Inequality and COVID-19 in Sweden: Relative risks of nine bad life events, by four social gradients, in pandemic vs. prepandemic years

**DOI:** 10.1073/pnas.2303640120

**Published:** 2023-11-09

**Authors:** Adam Altmejd, Olof Östergren, Evelina Björkegren, Torsten Persson

**Affiliations:** ^a^Swedish Institute for Social Research, Stockholm University, Stockholm 106 91, Sweden; ^b^Department of Finance, Stockholm School of Economics, Stockholm 106 91, Sweden; ^c^Department of Public Health Sciences, Stockholm University, Stockholm 106 91, Sweden; ^d^Aging Research Center, Karolinska Institutet, Stockholm 171 77, Sweden; ^e^Department of Economics, Stockholm University, Stockholm 106 91, Sweden; ^f^Institute for International Economic Studies, Stockholm University, Stockholm 106 91, Sweden; ^g^Suntory and Toyota International Centres for Economics and Related Disciplines, London School of Economics, London WC2A 2AE, United Kingdom

**Keywords:** COVID-19 pandemic, social gradients, multidimensional inequality, pandemic vs. prepandemic inequality

## Abstract

The COVID-19 pandemic struck societies directly and indirectly, not just challenging population health but disrupting many aspects of life. Different effects of the spreading virus—and the measures to fight it—are reported and discussed in different scientific fora, with hard-to-compare methods and metrics from different traditions. While the pandemic struck some groups more than others, it is difficult to assess the comprehensive impact on social inequalities. This paper gauges social inequalities using individual-level administrative data for Sweden’s entire population. We describe and analyze the relative risks for different social groups in four dimensions—gender, education, income, and world region of birth—to experience three types of COVID-19 incidence, as well as six additional negative life outcomes that reflect general health, access to medical care, and economic strain. During the pandemic, the overall population faced severe morbidity and mortality from COVID-19 and saw higher all-cause mortality, income losses and unemployment risks, as well as reduced access to medical care. These burdens fell more heavily on individuals with low income or education and on immigrants. Although these vulnerable groups experienced larger absolute risks of suffering the direct and indirect consequences of the pandemic, the relative risks in pandemic years (2020 and 2021) were conspicuously similar to those in prepandemic years (2016 to 2019).

## Significance

We uncover direct and indirect effects of the COVID-19 pandemic across multiple social groups. Compared to previous research, the value added is to derive harmonized estimates of multidimensional inequalities from high-quality population registers. So far, such estimates have been reported separately by different scholars, using hard-to-compare metrics and discipline-specific methods. By taking a bird's-eye view, our article unveils how unequally the pandemic hit, even in an egalitarian country like Sweden. We expose how different pandemic burdens fell heavier on disadvantaged groups—no matter whether we look along social gradients defined by income, education, or world region of birth. But our results also reveal a surprising inertia, with pandemic patterns of health and economic inequalities being very similar to prepandemic patterns.

During 2020, a new coronavirus swept across the world. The spread of the virus, together with mitigation and adaptation efforts by governments and individuals, transformed many aspects of life. The pandemic did not just harm human health but the healthcare system, the economy, and the ways people work and socialize.

Existing research focuses on one or a few consequences of the pandemic at a time and largely follows traditional disciplinary boundaries (for overviews, see refs. 1–5). Of course, highly specialized inquiries are essential to understand different dimensions of the pandemic, but relying on piecewise studies of how specific groups of individuals fared in terms of a single outcome can make us lose sight of the bigger picture. Moreover, to assemble one’s own overview by compiling and comparing evidence across disciplines is a daunting task due to diverging measures and methods. For the reader to better see the pandemic’s full impact, we complement narrowly focused research with an account that favors breadth and comparability over issue-specific nuance.

Our broad account emphasizes social inequalities. Setting aside cross-country differences, many aspects of health, wealth, and life chances are unequally distributed within countries. To provide a thorough picture, we thus consider a large set of outcomes and several, alternative criteria of social stratification.

By now, we know that severe morbidity and mortality in COVID-19 were more widespread among vulnerable groups (6). But this was not evident at the start of the pandemic. In the United States and Europe, affluent groups were among the first to be infected (7–9). Further, medical conditions may have less pronounced social gradients when less is known about prevention and treatment (10, 11). Still, public health scholars cautioned early on that disadvantaged groups were at risk of higher exposure to COVID-19, due to limited control of working and living conditions, as well as higher vulnerability to infections, due to lack of resources and preexisting health conditions (12).

Studies of multiple social inequalities during the pandemic are scarce. One notable study relies on publicly available survey data from multiple countries to document differences between men and women (13). But comprehensive accounts of several social gradients—long vs. short education, high vs. low income, or natives vs. migrants—do not seem to exist. Further, it is hard to paint a consistent picture from data that pertain to different populations, as the pandemic unfolded in such different ways across both time and place. We thus limit ourselves to analyzing how the pandemic affected multiple dimensions of inequality in a single country. Although Sweden may be considered an atypical case in some respects, the same can be said about any single population. Sweden constitutes an interesting instance, in that it was significantly hit in the early phase of the pandemic (14), when little was known about the virus and with little time to prepare. Moreover, the country has a wealth of administrative registers, which allow us to assess inequalities in several dimensions for the full population. The first of two research questions that we address is thus: (RQ1) Did the groups most severely hit by COVID-19 morbidity and mortality in Sweden also experience more severe indirect consequences of the pandemic?

Sudden external shocks can disrupt social inequalities, as these reflect long-term and interacting social, economic, and physiological forces. On the one hand, if pervasive disparities reflect such processes, a rattling event—like a war or pandemic—can potentially decrease these inequalities (15, 16). For example, processes that normally generate a heavier burden of somatic and psychiatric ill health in vulnerable groups could be disrupted by a sudden outside shock. Further, access to medical care may become more equal in a medical system, which is forced to prioritize emergency care over elective care, as resources are set aside to fight a new and overwhelming adversary (17). Almost by definition, individuals with abundant economic resources have more to lose (18). On the other hand, one can argue the opposite. For example, large shocks may magnify health inequalities, if lopsided resources make some people resilient and others exposed. As the pandemic struck the hardest against different sectors and occupations than other significant recessions have done—e.g., against restaurants and retail, rather than the financial sector—it could also have magnified or reduced existing economic inequalities.

The end result of such potentially countervailing forces—as well as of public policy interventions—is ambiguous. It is thus an empirical question whether social inequalities became wider or narrower during the pandemic. The second question we address is: (RQ2) Did the pandemic magnify or reduce existing inequalities in Sweden?

For both research questions, we study inequalities during 2020 as well as during 2021. In 2020, the first wave (and early second wave) of the pandemic struck Sweden hard and suddenly, when it came to health as well as other aspects of well-being. The following year entailed a gradual return to normal, with increasing immunity, a massive vaccination campaign, and an economic recovery.

To answer the two questions, we exploit a number of Sweden’s individual-level administrative registers. We use the information in these registers to estimate inequalities in the incidence of nine carefully chosen binary events. Three of these concern direct health consequences of the pandemic: COVID-19 death, COVID-19 hospitalization, and verified Severe Acute Respiratory Syndrome Coronavirus 2 (SARS-CoV-2) infection. Of course, they are observed only during the pandemic. The other events are observed both before and during the pandemic. Two of them represent strains on general somatic and psychiatric health: dying of any cause and visiting a psychiatric clinic, respectively. Two other events capture (lack of) access to medical care: nonsurvival 1 y after receiving a new cancer diagnosis and nonsurvival 30 d after undergoing surgery.^*^ Finally, we gauge economic hardship via two events: entering into unemployment and losing more than 1 mo worth of income. Together, these nine negative life events allow us to paint a diverse picture of different life burdens, before and during the pandemic.

Normally, these outcomes, and the problems they reflect, would be independently analyzed by researchers from different disciplines, using different statistical procedures. Because we define all outcomes as binary events, we can consistently compare them in terms of relative risks, which we estimate with the same statistical methods. Moreover, we study the relative risks along social gradients in four dimensions: gender, region of birth, education, and income. The common data sources allow us to estimate inequalities across our nine outcomes in these four dimensions for the same population.

## Results

Before describing the results that pertain to our main research questions, we set the scene by presenting descriptive statistics in *Background*.

### Background.

In this subsection, we first show inequalities in the relative risks of the six non-COVID events in prepandemic years 2016 to 2019. Then, we outline aggregate trends for all events for all years from 2016 to 2021.

#### 2016 to 2019 inequalities (Fig. 1).

Consider the six non-COVID outcomes over the prepandemic years 2016 to 2019. As mentioned, we estimate the relative risks for these bad life events across four dimensions: gender, region of birth, education, and income. We estimate the risk for each event and group as the (average) risk for the group relative to the (average) risk for the full population^†^. Thus, a value above (below) 1 indicates a higher (lower) risk than the population average. Regression coefficients behind our relative-risk estimates are presented in *SI Appendix*, Tables S8–S16. The underlying individual-level regressions control for age, to account for age composition across groups, and administrative region of residence (fixed effects), to allow for the influence of spatial variation in local labor markets and health systems across groups.

**Fig. 1.**
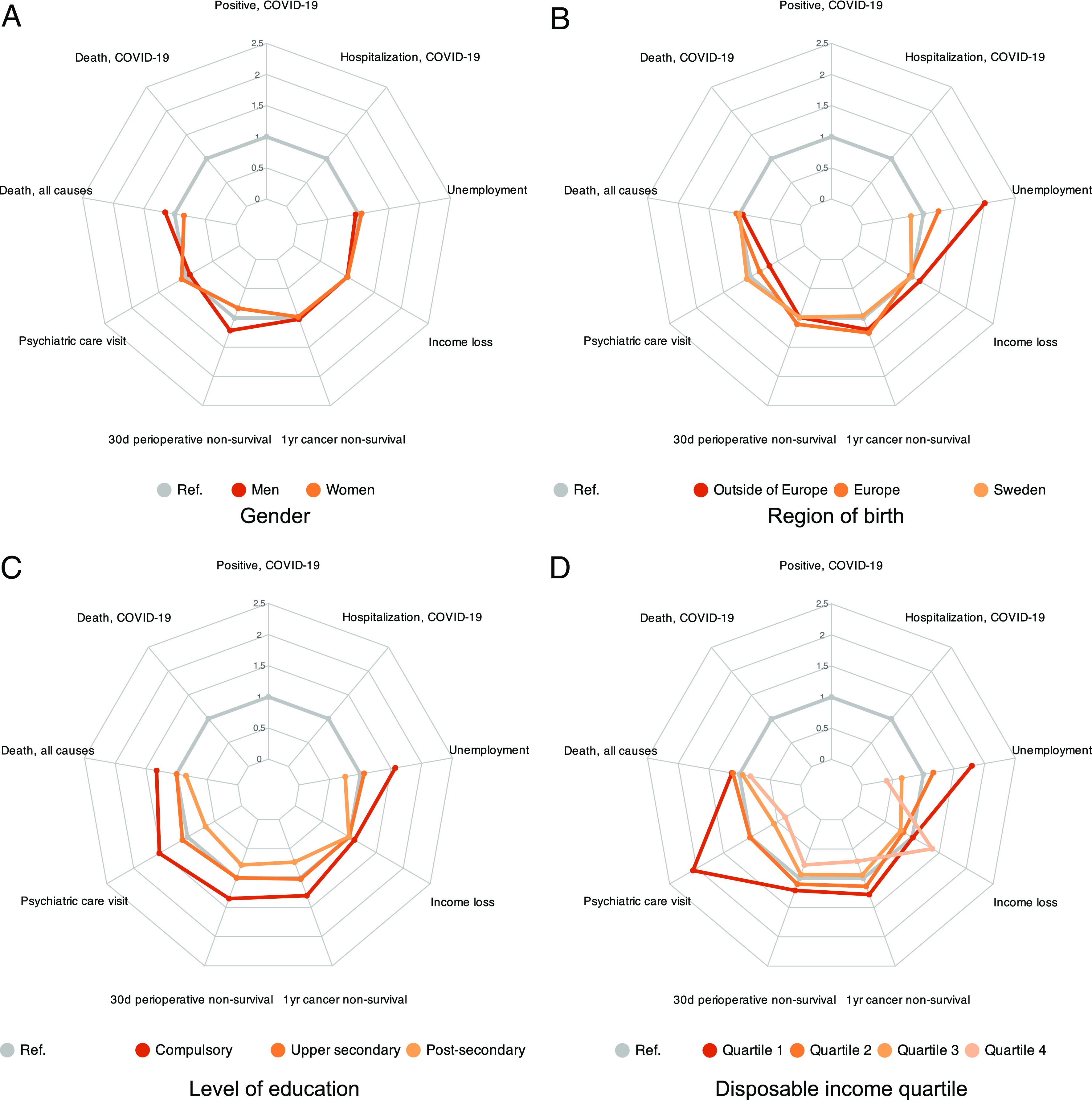
Multidimensional inequality, 2016 to 2019. Notes: The radar plots in the figure show relative risks for each of six negative life events in each of four dimensions, gender (panel *A*), region of birth (panel *B*), education (panel *C*), and income (panel *D*). The different-colored curves represent different social groups, as explained in the legend. Their respective relative risks are estimated in regressions that include controls for age and region. The figure shows these point estimates (reported with SEs in *SI Appendix*, Tables S8–S16), computed as predictive margins compared to population averages. Thus, a value of, say, 1.5 for a group means that this group is 50% more likely to suffer from the negative event, compared to the population at large. The measures of relative unemployment risk and of relative income-loss risk are calculated in the total population aged 25 to 64 y, while the other measures are calculated in the population of all individuals 25 y and older.

Fig. 1 shows the point estimates of group-wise relative risks by help of four separate radar plots, one for each social dimension.^‡^ To facilitate visual comparison with inequalities in the pandemic years in Fig. 3 below (and in *SI Appendix*, Fig. S1), we include blank results for the three COVID-19 events in the radar plots for the prepandemic period.

Fig. 1*A* reveals that the 2016 to 2019 relative risks were similar across genders, except that men had somewhat higher relative risks to die of all causes and not to survive up to 30 d after surgery, while women had somewhat higher relative risks of visiting an outpatient psychiatric care clinic. This could be because women and men partly suffer from different health problems and/or interact in distinct ways with the health system (19).

The gradients across different birth regions in Fig. 1*B* are more dramatic, at least on the economic side. What stands out from Fig. 1*B* are the relative risks of unemployment. These were two and a half times as high—2.00 vs. 0.80—for those 11% of the population born outside of Europe (see *SI Appendix*, Table S1 for the distribution of all groups) compared to natives, with the relative risk for immigrants born in Europe (another 11% of the population) in between. The risks of substantial loss of income were ordered in the same way but with much smaller differences. Relative risks of nonsurvival after operations and new cancer diagnoses were somewhat higher for Europe-born than for Sweden-born, as were the risks of dying of all causes and visiting a psychiatric clinic. However, the risks for the outside-Europe born were more similar to those for natives, or even lower in the case of psychiatric visits—the one exception being nonsurvival of new cancers. The low relative risks for distant migrants reflect the fact that they tend to have better health—sometimes even better than natives—probably due to positive health selection (20, 21).

As Fig. 1*C* clearly reveals, relative risks by education followed familiar social gradients for somatic and psychiatric health problems, differences in healthcare access, and economic strain. In fact, people with less than upper secondary education (17% of the population) suffered from the highest relative risks, and those with tertiary education (39%) the lowest relative risks, for all the six negative events. The steepest gradients were seen for unemployment, followed by psychiatric visits, nonsurvival after surgery, nonsurvival from new cancers, and death from all causes.

The relative risks by income in Fig. 1*D* follow similar, well-known gradients. Relative risks fall monotonically as we go from the lowest to the highest income quartile for five out of six negative life events, with the steepest gradients for psychiatric visits and unemployment. The one exception is that the risk of substantial income loss was at its highest in the top-income quartile; thus, the highest incomes were also the most volatile.

#### Aggregate outcomes 2016 to 2021 (Fig. 2).

**Fig. 2.**
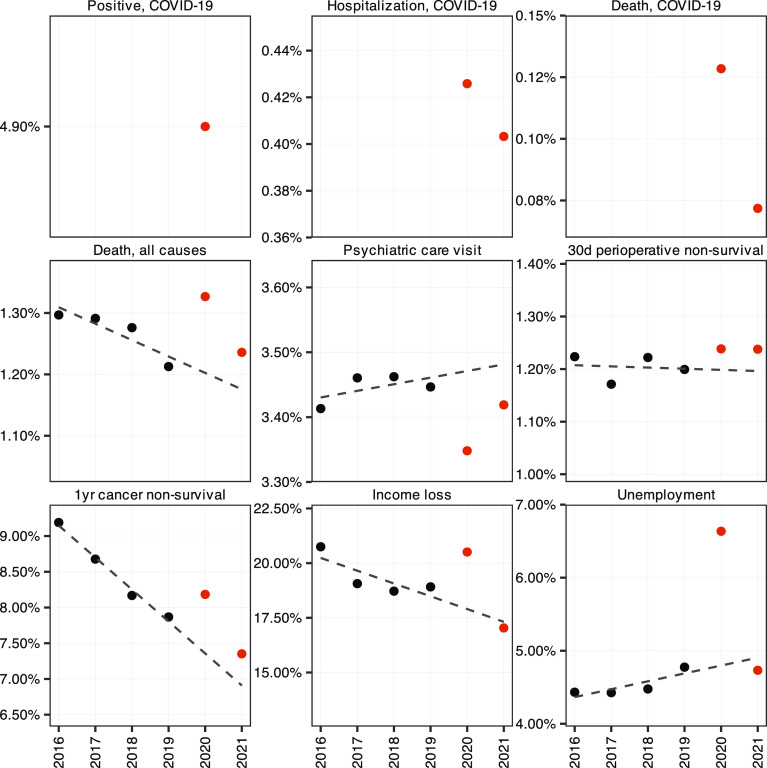
Population averages, 2016 to 2021. Notes: The figure shows population averages for each year in our sample. A linear regression line has been fit to the data points for 2016 to 2019 and extended to 2021. The risks of unemployment and of income loss are calculated in the population aged 25 to 64 y, while cancer and surgery mortality are based on the diagnosed or treated populations, respectively. The other outcomes are calculated in the population of all Swedes 25 y and older.

As a second part of the background, we show the evolution over time of the nine aggregate outcomes that we study. Fig. 2 plots the annual share of the whole population who experienced each negative event from 2016 to 2021. The first pandemic year, 2020, was remarkably different from 2016 to 2019 for all outcomes. The next pandemic year, 2021, was a little more diverse, but almost all outcomes (except for 30-d perioperative nonsurvival) returned toward prepandemic levels or trends. This is intuitive, as 2021 marked a transition from full-ranging pandemic, quarantine, and economic decline to ongoing infections but higher immunity, widespread vaccination, and economic recovery.

The top row in Fig. 2 shows that almost 5% of the entire population tested positive for SARS-CoV2^§^ at least once during 2020, that 0.43% of the population became sick enough in COVID-19 to be hospitalized, while 0.13% died from the infection. In 2021, the risks of hospitalization and death were 0.40 and 0.07%, respectively.

For general somatic health, the all-cause risk of death was more than 10% higher in 2020 than in 2019, a clear break with a declining mortality trend. Mortality decreased in 2021 but still stayed above the prepandemic trend. As for general psychiatric health, the share of the population who visited a psychiatric clinic declined by some 3% in 2020; in 2021, it remained below the slightly increasing trend observed before the pandemic. Other measures of psychiatric health—like new prescriptions of antidepressants and suicides—exhibit similar patterns before and during the pandemic (*SI Appendix*, Fig. S3). *Discussion* further discusses the difficulties of measuring mental health problems in the pandemic.

For nonaccess to health care in the population, the share who died within 1 y after a new cancer diagnosis went up by half a percentage point—almost 10%—when we compare 2020 to a downward prepandemic trend (22).^¶^ The lower survival rate is consistent with the notions that COVID-19 patients may have crowded out cancer screening and pushed new diagnoses toward more serious cases, or that fewer outpatient visits due to fears of COVID-19 infection may have led to fewer early diagnoses of treatable cancers. In 2021, the cancer survival rate reapproached its prepandemic declining trend. Fewer surgeries were performed in both 2020 and 2021 relative to previous years (23). Notably, this decline was proportional to the number of ongoing COVID-19 hospitalizations (*SI Appendix*, Fig. S2), strongly indicating that the capacity to treat medical problems was reduced by the pressure from the pandemic. In 2020, perioperative 30-d nonsurvival only went up marginally, which is consistent with declines mainly reflecting elective surgery, but emergency surgery staying at the prepandemic level (*SI Appendix*, Fig. S2). In 2021, nonsurvival remained unchanged, despite an overall smaller reduction in surgeries (24).

Finally, in the economic dimension, the aggregate unemployment rate went up sharply from 2019 to 2020. The share of people at working age who lost more than a month’s income also rose in 2020, relative to the trend of preceding years (although the share was on par with 2016). In 2021, as the economic crisis petered out, both economic outcomes returned toward more normal, prepandemic levels.

### Those Severely Affected by COVID-19 Also Suffered More from Other Negative Events in the Pandemic.

We now ask whether the groups who suffered severely from COVID-19 were also worse stricken by the indirect effects of the pandemic (RQ1). In *The Pandemic Did Not Substantially Alter Existing Inequalities*, we will change focus and ask how the social gradients for these indirect effects in the pandemic compare to prepandemic inequalities (RQ2).

**Fig. 3.**
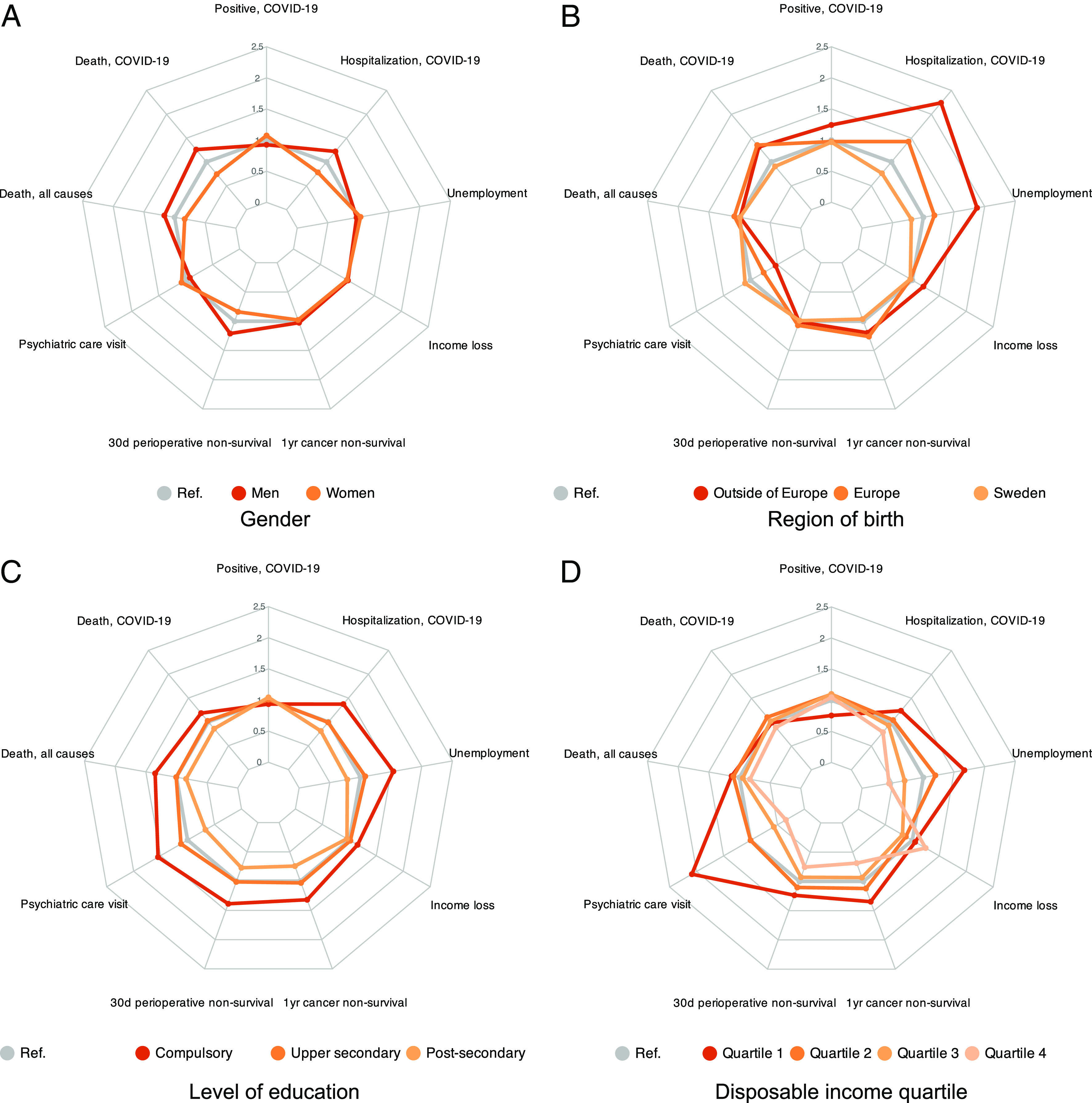
Multidimensional inequality, 2020. Notes: The radar plots in the figure show relative risks for each of nine negative life events in each of four dimensions, gender (panel *A*), region of birth (panel *B*), education (panel *C*), and income (panel *D*). The different colored curves represent different social groups, as explained in the legend. Their respective relative risks are estimated in regressions that include controls for age and region. The figure shows these point estimates (reported with SEs in *SI Appendix*, Tables S8–S16), computed as predictive margins compared to population averages. Thus, a value of, say, 1.5 for a group means that this group is 50% more likely to suffer from the negative event, compared to the population at large. The measures of relative unemployment risk and of relative income-loss risk are calculated in the total population aged 25 to 64 y, while cancer and surgery mortality are based on the diagnosed or treated populations, respectively. The other measures are calculated in the population of all individuals 25 y and older.

We begin by studying the pandemic inequalities in 2020. Fig. 3 shows the relative risks during 2020 in four radar plots analogous to those in Fig. 1. As before, the regressions that underlie the relative risks (predictive margins) control for age categories and regional indicators, where the latter now additionally capture differences in regional mitigation policies and local infection rates. Again, the estimated regression coefficients behind the relative risks in the figure are available in *SI Appendix*, Tables S8–S16.

#### Gender (panel 3*A*).

In 2020, men were more likely to be hospitalized or die from COVID-19, but women were more likely to test positive for the virus. In part, this reflects women being more likely to get tested (see refs. 25 and 26 and *SI Appendix*, Table S3). Similar to the prepandemic patterns, dying from all causes and not surviving surgery was more common for men, but visiting a psychiatric clinic was more common for women. The risks of unemployment and substantial income loss in the pandemic were similar across genders.

#### Region of birth (panel 3*B*).

Foreign-born individuals had a higher relative risk of hospitalization and death due to COVID-19 in 2020. The relative risk of admission to hospital with COVID-19 was close to 2.2 for those born outside of Europe and about 1.4 for those born in Europe. But the relative risks of COVID-19 mortality were reversely ordered, probably as a result of the better underlying health of distant migrants mentioned in connection with Fig. 1. The measures of general health and healthcare access look similar to their prepandemic counterparts (see further discussion in *The Pandemic Did Not Substantially Alter Existing Inequalities*). As for economic outcomes, migrants faced adverse gradients of unemployment and income loss in the pandemic, as they did in the prepandemic period.

In this dimension, what stands out the most about the 2020 relative risks are the pandemic effects among migrants from outside Europe, who faced large relative risks of COVID-19 hospitalization and of unemployment compared to natives. Moreover, the gradients in COVID-19 morbidity and mortality are substantially steeper than the gradients for general somatic and psychiatric health.

#### Education (panel 3*C*).

In 2020, individuals with lower education faced progressively higher relative risks of severe COVID-19 (hospitalization or death) but lower risks of testing positive. Conversely, higher-educated individuals had a lower relative risk of severe COVID-19 but a higher risk of testing positive. These gradients are similar in terms of direction, but have less slope, than those for different regions of birth. The same social gradients for health, nonaccess to health care, and economic strain as those we saw in the prepandemic period were observed during the pandemic as well.

#### Income (panel 3*D*).

Low-income households had a higher relative risk to suffer from severe morbidity and mortality in COVID-19 and of dying from any cause. The contrast between detected positive cases and risk of severe COVID-19 is again remarkable: The bottom income quartile was the most likely to be hospitalized but the least likely to test positive. As in the education dimension, we observe inequalities in the same direction before and during the pandemic in terms of general health problems, nonaccess to medical care, and risk of economic strain.

To summarize the results for RQ1, groups more likely to suffer from severe COVID-19 were also more vulnerable to other health issues and to economic difficulties. Migrants, and those with low education or income, saw higher relative risks of negative life events. The opposite is true for natives and those with high education or income. Gender patterns were more complex, with men at higher relative risk of severe negative health events. Almost paradoxically, the groups most likely to suffer severe COVID-19 in 2020 were the least likely to test positive. In *Discussion*, we further discuss this contrast between COVID-19 morbidity and positive tests.

We have thus far focused on the inequalities in 2020, the year when the first pandemic wave (and early second wave) struck quite dramatically against Sweden. The next pandemic year, 2021, saw the onset of the second and third waves. *SI Appendix*, Fig. S1 shows social inequalities for 2021 in a set of radar graphs, which are analogous to Fig. 3. This figure reveals that—the milder aggregate health and economic outcomes portrayed in Fig. 2 notwithstanding—the relative risks for all groups and outcomes in 2021 were very similar to those in 2020. In *The Pandemic Did Not Substantially Alter Existing Inequalities*, we delve further into this similarity.

#### Robustness.

Of course, individual group affiliations—especially, region of birth, education, and income—are not independent. Individuals in one worse-off group are also more likely to be part of other worse-off groups. *SI Appendix*, Tables S8–S16 also include regressions, where all group effects are jointly estimated. In these models, e.g., the group-level estimates for region of birth, hold education, income quartile, and gender constant. The main patterns are similar to those displayed in Fig. 3, although most group differences are smaller in the joint models. A notable exception concerns the relative risks for all-cause mortality by birth region: The mortality advantage of outside-Europe migrants is larger when gender, education, and income are held constant, confirming our earlier point that ceteris paribus far-away migrants have better health than others.

The results we have shown describe the incidence of negative events in 2020 by group. With individual-level data, one can also estimate the joint incidence of severe COVID-19 and other negative events by individual. *SI Appendix*, section B and Figs. S4 and S5 present additional results on interacted events: COVID-19 hospitalization together with either income loss or unemployment. Individual members of disadvantaged groups are more likely to experience a combination of severe COVID-19 and economic strain. This finding could be due to unobserved (to us) traits, which make people more vulnerable in both the health domain and the work domain.

### The Pandemic Did Not Substantially Alter Existing Inequalities.

Fig. 2 shows that most negative life events became more common in the full population in 2020 but started to return toward prepandemic trends in 2021. Fig. 3 and *SI Appendix*, Fig. S1 show that groups with higher relative risk of hospitalization and death from COVID-19 were more likely to suffer from other negative events, and in particular economic strain. But did the pandemic reduce or magnify the preexisting inequalities shown in Fig. 1?

To answer that question (RQ2), we use the results behind Figs. 1 and 3 and *SI Appendix*, Fig. S1 to juxtapose the relative risks for 2016 to 2019 vs. 2020 and 2021. Naturally, we can only do so for the six non-COVID-19 events. Inspired by earlier research on US mortality in ref. 27 plots relative risks during the pandemic (on the *y* axis) against the prepandemic relative risk in 2016 to 2019 (on the *x* axis). The 2020 relative risks are marked by colored dots, and the 2021 relative risks by colored crosses. Markers above (below) the diagonal (45-degree line) thus indicate higher (lower) risks during the pandemic than in the preceding 4 y. The shaded areas represent 5% (darker shade) and 10% (lighter shade) intervals around the prepandemic relative risk. The four parts of the figure rely on the same group divisions as Fig. 3, by gender, region of birth, education, and income.

**Fig. 4.**
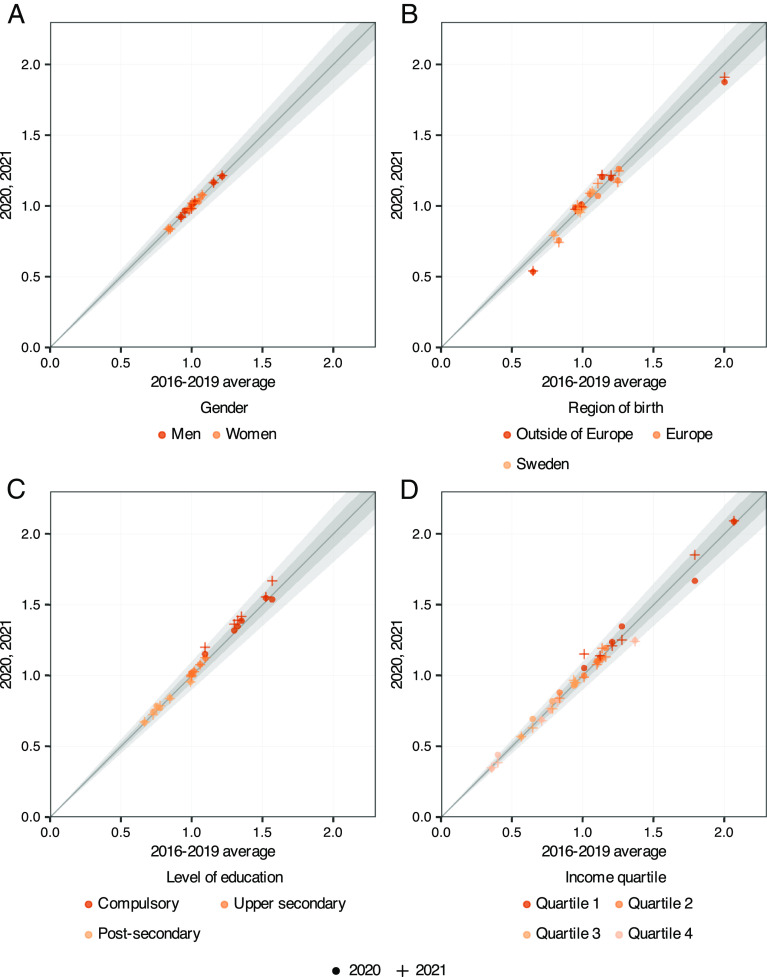
Relative risks 2020 and 2021 vs. 2016 to 2019. Notes: The figure shows relative risks for suffering each of the indirect negative events (where a 2016 to 2019 average can be calculated) in each of the four social dimensions panels (*A*–*D*). Each panel compares the risk for 2020 (dots) and 2021 (crosses), on the *y* axis, to the same risk averaged over the period 2016 to 2019 (on the *x* axis). For points on the diagonal, the relative risk of suffering a certain negative event before the onset of the pandemic is equal to the relative risk during the first or second pandemic year. The shaded areas indicate relative differences over time of 5% (dark) and 10% (light), respectively. The measures of relative unemployment risk and of relative income-loss risk are calculated in the total population aged 25 to 64 y, while cancer and surgery mortality are based on the diagnosed or treated populations, respectively. The other measures are calculated in the population of all individuals 25 y and older.

Strikingly, nearly all markers in all four graphs lie very close to the diagonal. Despite the substantial absolute impact of the pandemic on most negative life events in 2020, the relative risks of experiencing them almost did not change at all. In fact, only 2 out of a total of 72 (dot) markers for 2020 have their centroid outside the 10% cone, and only about a dozen outside the 5% cone. Among 2021 inequalities, only 3 out of 72 (cross) markers are outside the 10% cone, and another dozen outside the 5% cone.^#^ In the following, we comment further on these patterns and point to a few exceptions.

#### Gender (panel 4*A*).

The relative risks for men and women were very similar in both 2020 and 2021 relative to previous years.

#### Region of birth (panel 4*B*).

Most relative risks by regions of birth were approximately constant across prepandemic and pandemic years. Although migrants suffered from a higher overall death rate in COVID-19 than natives, the pandemic only marginally impacted relative risks for all-cause deaths by region of birth. We also observe the same relative risks in somatic and psychiatric health (dying from all causes, and visits to psychiatric care) during pandemic and prepandemic years. This is also true for the two measures of access to health care. Thus, and unlike their higher risks of COVID-19 morbidity and mortality, the foreign-born did not suffer disproportionately from the indirect health effects of the pandemic. Neither did their relative risks of economic strain go up significantly in the pandemic.^||^ Exceptions to the stable pattern of relative risks concern the share of outside-Europe, and Europe migrants who visited a psychiatric clinic, which fell further from already low levels—see the red and dark-yellow dots and crosses just outside and inside the 10% cone at the bottom left.

#### Education (panel 4*C*).

Inequalities in relative risks along the educational gradient in 2020 were very close to those in earlier years. Three modest exceptions are that, during the pandemic, the least educated saw a slight drop in their relative risk of unemployment but a small rise in their relative risks of all-cause mortality and major-income loss—see the red dots slightly off the diagonal in the upper right of the figure (but well inside the 10% cone). In 2021, there was a tendency to larger inequalities by education in all-cause mortality, cancer survival, income loss, and unemployment—the red crosses off the diagonal to the upper right (but still well inside the 10% cone). The figure thus, tentatively, indicates that individuals with only compulsory education may experience a slower recovery from the pandemic shock.

#### Income (panel 4*D*).

In this dimension as well, relative risks were basically stable. Again, one exception concerns relative unemployment risk, which went down from 1.79 to 1.67 for the lowest quartile in 2020 but went up to 1.85 in 2121 (*SI Appendix*, Table S7). The same group experienced a gradual increase in the risk of relative income loss, from 1.01 before the pandemic to 1.05 in 2020 and 1.15 in 2021. Conversely, for the highest income quartile, the relative risk of major income loss fell—see the light-yellow dot and cross below the diagonal in the upper middle of the figure, but in 2020, the risk for unemployment for the group increased by 10%, only to fall back in 2021.

While we have spent some time discussing exceptions, this should not overshadow the main message from Fig. 4. In particular, the relative risks of negative life events in the first and later waves of the pandemic were remarkably stable, compared to prepandemic relative risks. In this sense, the inequalities in a variety of outcomes, and along multiple social gradients, turned out to be very stubborn, even in the wake of a new, large, and sudden shock and its aftermath.

However, when interpreting this bottom-line result, it is important to recall from Fig. 2 that the aggregate risk of most negative life events went up during the pandemic, especially in 2020. As relative risks stayed more or less constant, absolute risks—by definition—went up by more for disadvantaged groups (*SI Appendix*, Tables S4–S7 show both relative and absolute risks over both periods). In absolute terms, the pandemic thus led to more inequality: a larger number of deaths, higher unemployment spells, more frequent income losses, and a larger number of people who did not survive their cancer or surgery, in Sweden’s migrant population and among its least-educated and poorest residents.

## Discussion

This paper shows unique evidence on multidimensional inequality during Sweden’s COVID-19 pandemic, along four different social gradients. We consider nine diverse outcomes, which are rarely considered together and in the same statistical framework, given existing disciplinary boundaries. With few exceptions, direct and indirect burdens of the pandemic fell heavier upon more disadvantaged social groups.

While the results paint a comprehensive picture of pandemic and prepandemic inequalities, the broad-brush strokes conceal five facets of important detail.

First, our positive-case measure reflects both disease incidence and test frequency. The propensity to order a test was lowest among those most hit by severe disease (*SI Appendix*, Table S3). It turns out that cancer has a similar inverse pattern (*SI Appendix*, Table S2), whereby groups with higher death risk are less likely to get diagnosed. The same decline in healthcare access may thus have hit disadvantaged groups harder, due to more undetected health problems already before the pandemic. The inverse patterns also suggest potential flaws in existing COVID-19 research that relies on detected cases to measure contagion or to calculate case-fatality rates.

Second, estimated inequalities refer to nationwide groups and yearly data. While the groups we have considered reflect essential sources of stratification, a great deal of heterogeneity remains within these groups. For example, more exposed occupations were a strong determinant for the risk of serious COVID-19 (28), and such exposed jobs are held by members of all groups that appear in the paper (even though disadvantaged groups more likely work in highly exposed occupations). Similarly, risks varied regionally and seasonally, with the reach and severity of SARS-CoV-2 infections. More granular analyses would likely add valuable nuance to the broad picture presented here.

Third, some of our measurements are crude. In particular, gauging mental health problems by visits to outpatient psychiatric clinics is, at best, a rough approximation of population mental health, though we find similar overall patterns in prescriptions of antidepressants and suicides (*SI Appendix*, Fig. S3). In part, these measures may also reflect that some groups tended to avoid care or found it more difficult to access. That being said, it is unclear how the pandemic affected mental health (29, 30), as it altered detrimental (unemployment and social isolation) as well as protective (lower work stress and less time away from family) drivers of mental health. Moreover, since the pandemic was a large shock and transformed daily lives in several ways, it is difficult to interpret what might have happened by referring to evidence on mechanisms, which was collected in more normal, less comparable times.

Fourth, the nine events considered in the paper capture important direct and indirect effects of the pandemic. But they leave out crucial and potentially unequal outcomes. Examples include lower subjective well-being, limited care for those needing daily assistance, and forfeited human capital due to restricted schooling.

The schooling point illustrates a fifth limitation of the paper: its focus on short-term inequality. Long-term footprints of negative events—say, canceled doctor’s appointments, unexpected unemployment spells, or sudden income losses—may be more serious for groups with fewer resources for self-insurance. An indication of this may be a continued elevation of perioperative nonsurvival in the later stages of the pandemic, despite a return toward prepandemic rates of performed surgeries. This is a possible indication of less severe complications going untreated in 2020 and developing into severe cases in 2021. Moreover, our findings mildly suggest that some groups found it more difficult to recover from the pandemic than others, namely low-income individuals in terms of economic conditions, and low-educated individuals in terms of economic conditions and health. How societies and individuals will manage to recover may thus shape postpandemic social inequalities.

Understanding the challenges and—more generally—the broad consequences of the past pandemic, or future pandemics, for different groups in society calls for scientific collaboration across disciplines. Since pandemics can be described as biological processes operating on social networks, that collaboration should draw on rich data and rely on complementary insights by scholars in medical and social sciences. In this paper, we have just scratched the surface of that research agenda.

Our most surprising finding is that the relative risks across social groups of being hit by negative events were strikingly similar in the pandemic and in the 4 y preceding it. In our view, it is remarkable how path-dependent inequalities in health, wealth, and access to health care turned out to be, even following a large, unusual, unexpected, and arguably exogenous shock like the COVID-19 pandemic.

## Materials and Methods

The individual-level data used in this paper were retrieved from a variety of Swedish registers kept by different public agencies, such as Statistics Sweden, the Public Health Agency of Sweden, the National Board of Health and Welfare, and the Swedish Unemployment Service. We obtained permission to use this information for the research described in the paper from Sweden’s Ethical Review Authority (permit numbers 2021-02225, 2022-013550-02, and 2022-06118-02). The data are classified as secret, meaning that we cannot publicly share it, on legal and ethical grounds. However, all computer code used for processing and analysis is available online (31). To validate or extend this work, interested researchers can thus (subject to ethical review) obtain the same material from the indicated register holders and reproduce our results using the provided code.

The analysis in the paper is based on Swedish full-population register data for the years 2015 to 2021. The dataset includes all registered residents in the country, aged 25 and older, for a total of 7,968,619 unique individuals. In the analyses of unemployment and income, the population is limited to individuals of working age (25 to 64), excluding about 2 million individuals. Nonsurvival from cancer and surgeries are calculated from the populations that were diagnosed with cancer (N = 483,343) or underwent surgery (N = 4,006,464), respectively. These populations are divided into groups across four sociodemographic facets: registered gender, world region of birth (Sweden, Europe, or outside Europe), highest attained level of education (below high school, high school, and tertiary education), and disposable income (by quartiles of disposable income).

To compare very different outcomes on a common scale, we define all outcomes as binary events—this allows us to estimate relative risks for different groups. Some of these binary events capture direct consequences of the COVID-19 pandemic: confirmed infections, hospitalizations, and deaths from COVID-19. Others capture indirect consequences; on health: all-cause deaths and visits to psychiatric outpatient clinics; on access to medical care: nonsurvival 1 y after receiving a new cancer diagnosis, and nonsurvival 30 d after undergoing surgery; and on economic strain: entries into unemployment, and losses of at least 1 mo worth of disposable income between two successive years. Some of these outcomes are proxies, but we judge them to be among the best measures available in the registers.

The precise definition of each population, subgroup, and outcome measure is stated in *SI Appendix*, section A. There, we also discuss the advantages and disadvantages of these specific metrics of the pandemic’s direct and indirect consequences.

For each sociodemographic group, we compute the relative risks of experiencing each separate negative binary event from statistical estimates based on OLS regressions, which take into account the joint distribution of individual socioeconomic traits in each group. The reported relative risks are calculated as the predictive margin for each group to experience a particular event in the specific period (2016 to 2019, 2020, or 2021), compared to the average predictive margin for the same period in the entire population.

## Supplementary Material

Appendix 01 (PDF)

## Data Availability

Computer code used for processing and analysis data have been deposited in a code repository (31). The individual-level data used in this paper were retrieved from a variety of Swedish registers kept by different public agencies, such as Statistics Sweden, the Public Health Agency of Sweden, the National Board of Health and Welfare, and the Swedish Unemployment Service. We obtained permission to use this information for the research described in the paper from Sweden’s Ethical Review Authority (permit numbers 2021-02225, 2022-013550-02, and 2022-06118-02). The data are classified as secret, meaning that we cannot publicly share it, on legal and ethical grounds. However, all computer code used for processing and analysis is available online. To validate or extend this work, interested researchers can thus (subject to ethical review) obtain the same material from the indicated register holders and reproduce our results using the provided code.

## Footnotes

^*^These are established indicators for the quality of cancer care and for the access to safe anesthetic and surgical care, respectively (see the *SI Appendix, Materials and Methods* section).

^†^Risks are estimated by average predictive margins (also called average adjusted predictions). We calculate the average predictive margins for a certain event, group, and year, from the estimated model in two steps: i) predict the probability that each individual in the population will experience the given event in the given year, keeping age and region of residence at their observed values, but treat everybody as a member of the given group, and ii) average over these individual predicted probabilities. We provide more detail in *SI Appendix*, section A.4.

^‡^The figure does not include any CIs—note that the relative risks are not based on data from a sample, but from the full Swedish population. SEs, clustered at the individual level, are still included in *SI Appendix*, Tables S8–S16. The implied CIs are too narrow to be visible in the graphs.

^§^We do not have access to individual-level data on confirmed infections for the year 2021. According to aggregate data publicly available from the Public Health Agency of Sweden, there were 384,203 confirmed infections in 2020 and 963,597 in 2021 (24). But these numbers are difficult to compare both because the availability of testing was much higher in 2021 and because the time span used to define reinfection changed over time.

^¶^The observed pattern reflects longer secular trends of higher cancer incidence and lower cancer mortality. Our nonsurvival measure is based only on those individuals who did not die from COVID-19 and whose newly detected cancer was either an underlying or contributing cause of death.

^#^The observations outside—typically, marginally outside—the 10% cone are the risk of visiting psychiatric care for those born outside of Europe (relative risk goes from 0.65 in 2016 to 2019 to 0.54 in 2020) and the risk of unemployment in disposable income quartile 4 (0.40 to 0.44) in 2020. In 2021, they are psychiatric-care visits for the European-born (0.83 in 2016 to 2019 to 0.74 in 2021) and the outside-of-Europe-born (0.65 to 0.54), as well as the risk of income loss in quartile 1 (1.01 to 1.15). For the full set of relative risks, see *SI Appendix*, Tables S4–S7.

^||^It should be noted that the absolute increase in unemployment risks in 2020 was much higher for non-European immigrants, increasing from 9.06 to 12.45% (by 3.39 percentage points), than for native Swedes, who saw an increase from 3.60 to 5.34% (by 1.74 percentage points), see *SI Appendix*, Table S5.
