# Supplementary material for: Inequality and COVID-19 in Sweden: Relative risks of nine bad life events, by four social gradients, in pandemic vs. prepandemic years
Source: Proc Natl Acad Sci U S A. 2023 Nov 9;120(46):e2303640120. doi: 10.1073/pnas.2303640120 (PMC10655217; doi:10.1073/pnas.2303640120)
Supplement: Supplementary file 1 — Appendix 01 (PDF) [file pnas.2303640120.sapp.pdf]

Supporting Information for:

Inequality and COVID-19 in Sweden: Relative risks of nine bad life events, by four social gradients, in pandemic vs. pre-pandemic years

Adam Altmejd[*,†] Olof Östergren[‡,§] Evelina Björkegren[¶] Torsten Persson[‖,**]

Corresponding author: Adam Altmejd adam.altmejd@sofi.su.se.

This PDF file includes:

- Supporting text
- Figures S1 to S5
- Tables S1 to S16

[*]Swedish Institute for Social Research, Stockholm University
[†]Department of Finance, Stockholm School of Economics
[‡]Department of Public Health Sciences, Stockholm University
[§]Aging Research Center, Karolinska Insitutet
[¶]Department of Economics, Stockholm University
[‖]Institute for International Economic Studies, Stockholm University
[**]STICERD, London School of Economics

A Materials and methods

Population-wide administrative registers are a key feature of the Nordic administrative model. Different administrative registers hold longitudinal information on the demographic, geographic, social, economic, and health characteristics of each individual in Sweden's population. Permission to use this information for the research described in the paper was obtained from the Swedish Ethical Review Authority (permit 2020-06492 / 2021-01115 / 2022-01355-02).

A.1 Population definition

The analysis is based on the full population aged 25 years and older that were registered as residing in Sweden on the last day of 2015, 2016, 2017, 2018, 2019 or 2020. This population comprises 7,968,619 unique individuals. The data for each year of study includes the registered population aged 25 and older at the end of the previous year. For unemployment and income, the population is further limited to those between the ages of 25 and 64, comprising 5,841,729 unique individuals. For 1 year cancer non-survival and 30 day perioperative non-survival individuals enter the population at risk once they receive a new cancer diagnosis (483,343 unique individuals) or undergo surgery (4,006,464 unique individuals), respectively.

A.2 Group definitions

The population is divided into groups by four different categorizations. The data used to create them comes from Statistics Sweden's longitudinal database, LISA, which includes harmonized yearly information about all individuals in Sweden. Group categories in a certain year reflect information from the preceding year about each individual's registered gender, highest attained level of education, income, and region of birth. Disposable-income quartiles are calculated separately by birth cohort.

A.3 Outcome definitions

The study concerns nine negative, direct and indirect, events related to the pandemic. These events are defined from individual-level register information from Statistics Sweden, the Public Health Agency of Sweden, the National Board of Health and Welfare, and the Swedish Unemployment Service. The observation period is limited to the year 2020 in order to abstract from the effects of vaccinations (the Swedish COVID-19 vaccination program started in the first month of 2021). Since vaccine uptake also exhibits clear socio-economic gradients, it will influence many of the other measures. Studying the first year (and the first two waves) of the pandemic, nullifies the effect of these differences. Additionally, we do not have access to data unemployment episodes and cancer diagnosis after 2020.

Positive cases are defined by laboratory (PCR) confirmed infections of SARS-CoV-2. During the pandemic, COVID-19 was classified as a disease that is a threat to society—all confirmed cases of such a disease must, by law, be reported to the Swedish Public Health Authority and registered in the SmiNet register. However, this register only contains infections that were confirmed by a test. During 2020, testing capacity varied—both across regions and time—as did official priorities for using the scarce capacity.

The testing behavior also varied across groups. In general, groups that were the most likely to develop a serious infection were the least likely to order a test (SI Table S3).

Hospitalizations in COVID-19 are defined by at least one episode of inpatient care, with COVID-19 as the main diagnosis for admission, according to the Inpatient Care Register kept by the National Board of Health and Welfare. We include patients with both laboratory-confirmed tests (denoted by U07.1 in ICD-10) and those diagnosed by a physician without a test (U07.2). Physician-diagnosed cases were more prevalent in the first wave of the pandemic, when testing capacity was limited also in hospitals.

Deaths by COVID-19, as well as deaths from all causes, are identified from the Cause of Death register kept by the National Board of Health and Welfare. According to WHO guidelines, COVID-19 should only be recorded as a cause of death when the disease played a casual role in the events leading to death. Deaths from COVID-19 were defined by any deaths that included U07.1 or U07.2 as either the underlying (89.8 percent of deaths) or contributing (10.2 percent of deaths) cause of death. Deaths from any cause are defined as the individual dying during the specific year.

We identified visits to psychiatric clinics in specialized outpatient care. These are clinics where individuals can receive care for mental and behavioral disorders by a physician at a clinic, rather than a hospital, and can be identified by the first digit of the MVO (medicinskt verksamhetsområde, medical field of activity) code in the Outpatient Care Register kept by the National Board of Health and Welfare. These include clinics that specialize in a diverse set of treatments for mental and behavioral disorders including general and specialized psychiatry, gerontological psychiatry, alcohol- and substance abuse clinics, family counselling and forensic psychiatric care. We identified all individuals that made at least one such visit in a given year. Access to health care was made more difficult during the pandemic, in part because individuals purposefully avoided seeking care out of fear of getting infected or transmitting the virus to others. Those experiencing fear and anxiety, and thereby at an increased risk of needing psychiatric care, are also more likely to avoid contact with the medical system. In addition to visits to a psychiatric clinic we identify two additional indicators of mental health. Antidepressant use is defined from the Medical Drug Register kept by the National Board of Health and Welfare; specifically, from individuals who made at least one purchase of prescription anti-depressants (ATC: N06A). To capture new instances of poor mental health, as opposed to ongoing spells, the definition includes only individuals who made no such purchase in the previous calendar year. We identified deaths from intentional self harm (ICD-10: X60-X84) or injuries and poisonings of undetermined intent (ICD-10: Y10-Y34) in the Cause of Death Register kept by the National Board of Health and Welfare. These three measures indicate poor mental health indicate poor mental health of varying degrees of severity. All three measures indicate a decline in poor mental health in 2020 (Figure S3).[1]While the analysis focuses on the first wave, mental problems may have expanded as the pandemic progressed.

Patient survival after receiving a cancer diagnosis is an indicator of the quality of cancer control and captures the ability of the medical system of detecting and treating cancer in the population [3]. We identified all new cancer diagnoses (ICD-10: C00-C96) being reported to the National Cancer Register, kept by the National Board of Health and Welfare, and estimated vital status 365 days later using the Cause of Death register. We excluded patients that died from Covid-19 and patients where the cancer that was diagnosed was not reported as an underlying or contributing cause of death. Although this measure only capture health care access in one type of disease, other national registers on health-care consumption only cover inpatient care and visits to physicians in specialized outpatient care. No national register keeps track of individual visits in primary health care. However, every health-care provider must report any detected cancer to the cancer register, regardless of where and how it is detected. Even though

1. Whatever the quality of the measure, it is not clear that the pandemic has had an impact on population-level mental health. Findings in the literature have so far been mixed (see e.g. [1, 2]).

it includes only one type of disease, the cancer register thus provides a comprehensive indicator of the medical system's diagnostic capability, which is not available for other diagnoses.

The risk of death within 30 days of a surgical procedure, sometimes referred to as the perioperative mortality rate, is an established quality indicator of surgical care and anaesthesia [4]. We estimate the individual risk of dying within 30 days of surgery by first identifying all surgical procedures, either in inpatient care or in specialized outpatient care. These are identified by the KVÅ (classification of care measures) coding standard used in the Swedish Outpatient and Inpatient Care Registers. Each procedure is classified as either medical or surgical and registered by the day it is performed. We then use the Cause of Death Register to classify the individual vital status 30 days later. While this estimate is a quality indicator of medical treatment, it does not capture non-fatal complications of compromised quality or reduced access to medical care.

Unemployment is defined by having registered as unemployed at least once at the Swedish Unemployment Service during the year. It is necessary to register as unemployed in order to claim unemployment benefits, including private unemployment insurance (A-kassa). We only count new registrations.

Income loss is defined by having an annual disposable income, which is at least 8.3 percent lower than the year before, equivalent to losing one month's pay. Disposable income is defined as the individual income after taxes and transfers as declared on the individual tax statement and reported to the Tax and Income Register kept by Statistics Sweden.

A.4 Statistical models

All estimates are based on OLS regressions, the results of which are reported in Tables S8 to S16. For non-COVID-19 outcomes, the regressions include individual-year observations for each year between 2016 and 2020. Group membership is then interacted with an indicator variable indicating if the year is 2016–2019, 2020, or 2021—producing the difference between the pre-pandemic average and 2020, and 2021, respectively. Each regression includes binary indicators (fixed effects) for Swedish administrative regions, as well as 19 age categories interacted with the three period indicators. The standard errors are clustered at the individual level. Where applicable, the same procedure is used to produce supplementary analyses.

From these OLS regressions, predictive margins were calculated for every group in the population using Stata's margins command. This procedure generates the average predicted probability for a member of a certain group to experience a certain event, and is calculated by letting all observations belong to the group (keeping other covariates at their observed levels), using the estimated model to predict the outcome, and then average over these predictions. Relative risks are calculated by dividing the predictive margins for each group with the population average.[2]

A.5 Data and code availability

The individual-level data used in this study was retrieved from a variety of Swedish registers and is covered by public secrecy. Therefore, the data cannot be publicly shared, on legal and ethical grounds. However, all computer code used to process and analyze this data is available at `https://osf.io/yrju3/`. To

2. Computing the relative risks from estimates with alternative statistical models, such as logistic regression, produces similar results (not reported).

verify the analysis in the paper, interested researchers can thus (subject to ethical review) obtain the same material from the indicated register holders.

B Interactions of outcomes

In Figures S4 and S5 the risk of suffering from multiple negative events are displayed. Here we study hospitalization risk and either unemployment or income risk. Each panel shows the gradient of one of the four dimensions, and the two leftmost bar groups in each plot show the same relative risks as in the main radar plots. The third bar group however shows the relative risk for the interacted outcome — that the same individual suffers from both events. To understand what it means that these interacted effects are always larger for the worst-off groups, consider the following example. If only exactly those who are hospitalized for COVID-19 became unemployed, all gradients would look the same. If instead there was no overlap between hospitalizations and unemployment, there would be no gradient at all visible in the third bar group. What we see is that the relative risks for the worst-off groups are usually higher. This means that it is more common in these groups that COVID-19 hospitalization and unemployment or income loss go together. While this happens in all groups, those worst-off have a weaker connection to the labor market and their hospitalization is more likely to cause further negative shocks.

References

1. G. Prati, A. D. Mancini, The Psychological Impact of COVID-19 Pandemic Lockdowns: A Review and Meta-Analysis of Longitudinal Studies and Natural Experiments. *Psychological Medicine* **51**, 201–211 (2021).
2. Z. Van Winkle, E. Ferragina, E. Recchi, The Unexpected Decline in Feelings of Depression among Adults Ages 50 and Older in 11 European Countries amid the COVID-19 Pandemic. *Socius* **7** (2021).
3. A. Verdecchia *et al.*, Patient Survival for All Cancers Combined as Indicator of Cancer Control in Europe. *European Journal of Public Health* **18**, 527–532 (2008).
4. D. A. Watters *et al.*, Perioperative Mortality Rate (POMR): A Global Indicator of Access to Safe Surgery and Anaesthesia. *World Journal of Surgery* **39**, 856–864 (2015).

C Supporting figures

Figure S1. Multidimensional Inequality, 2021

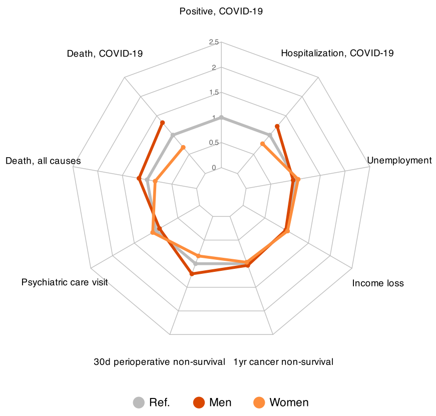

(a) Gender

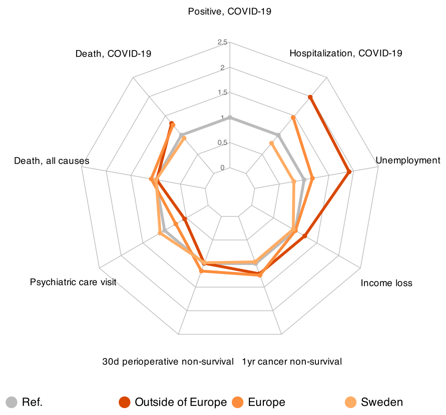

(b) Region of birth

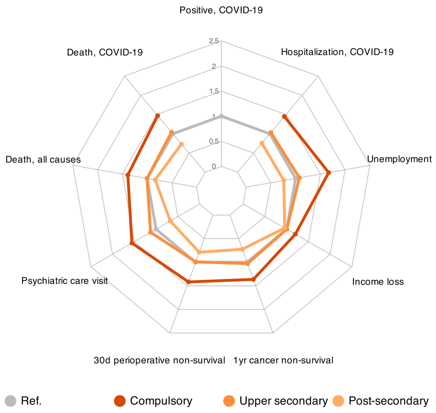

(c) Level of education

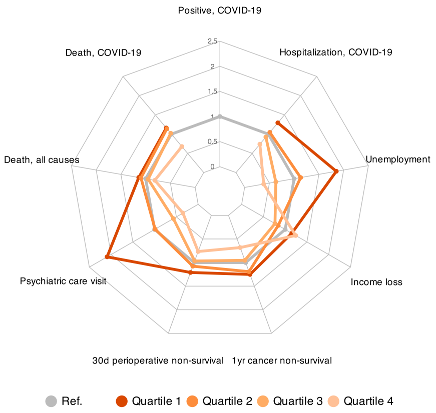

(d) Disposable income quartile

Notes: This figure is analogous to Figures 1 and 3 in the main text but reports relative risks for 2021.

Figure S2. Performed elective and emergency surgeries relative to the maximum capacity

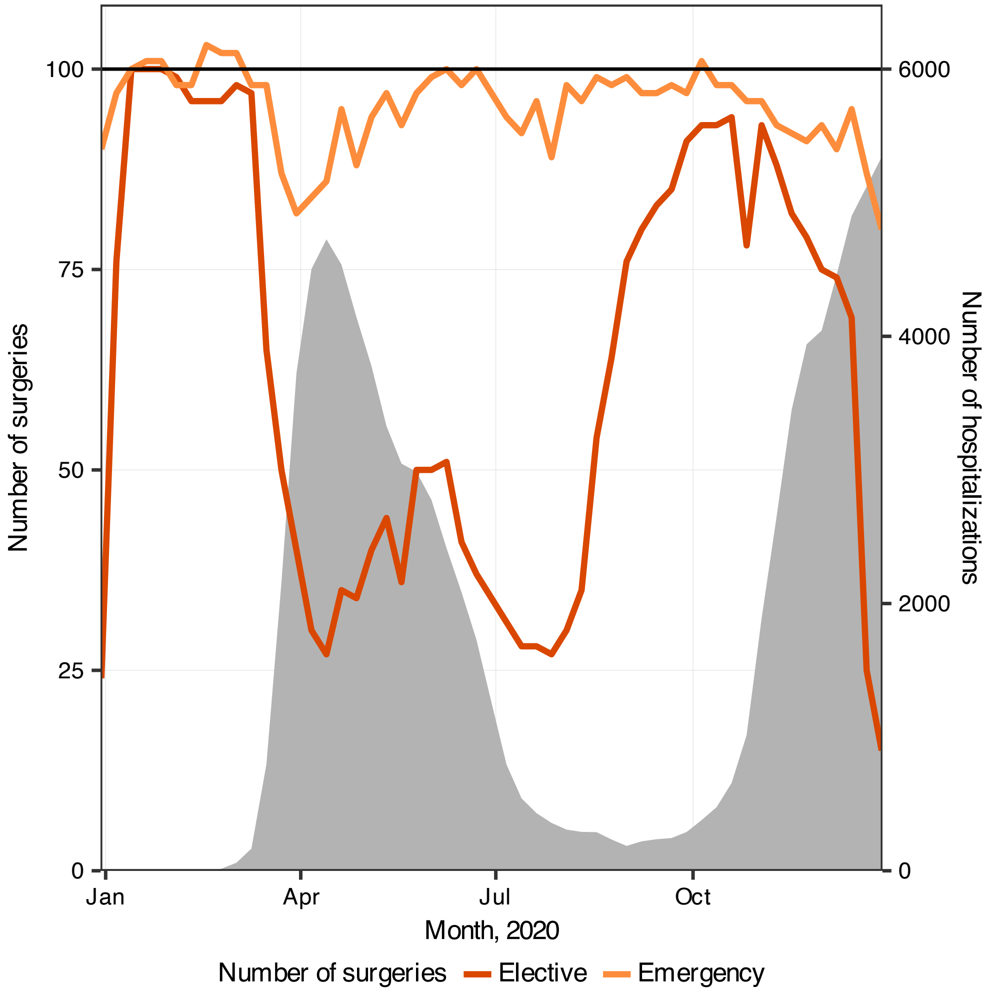

Notes: The numbers of elective and emergency surgeries per week is are reproduced from the numbers reported by Svenskt Perioperativt Register. They are reported in relation to the maximum capacity (8400 elective and 2900 emergency surgeries) which is set to 100. The number of ongoing COVID-19 hospitalizations were calculated using the inpatient care register and give an indication of the burden of COVID-19 on the medical system.

Figure S3. Population averages for additional psychiatric outcomes

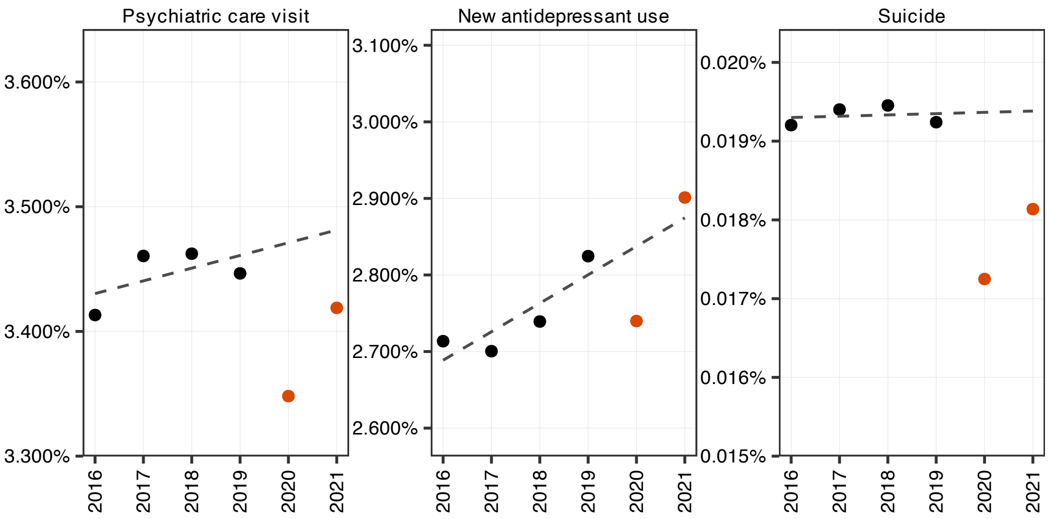

Notes: The figure shows population averages for each year in our sample. A linear regression line has been fit to the data points for 2016-2019 and extended to 2020. The outcomes are calculated in the population of all Swedes 25 years and older.

C.1 Interaction figures

Figure S4. Individual interaction: Hospitalization and unemployment

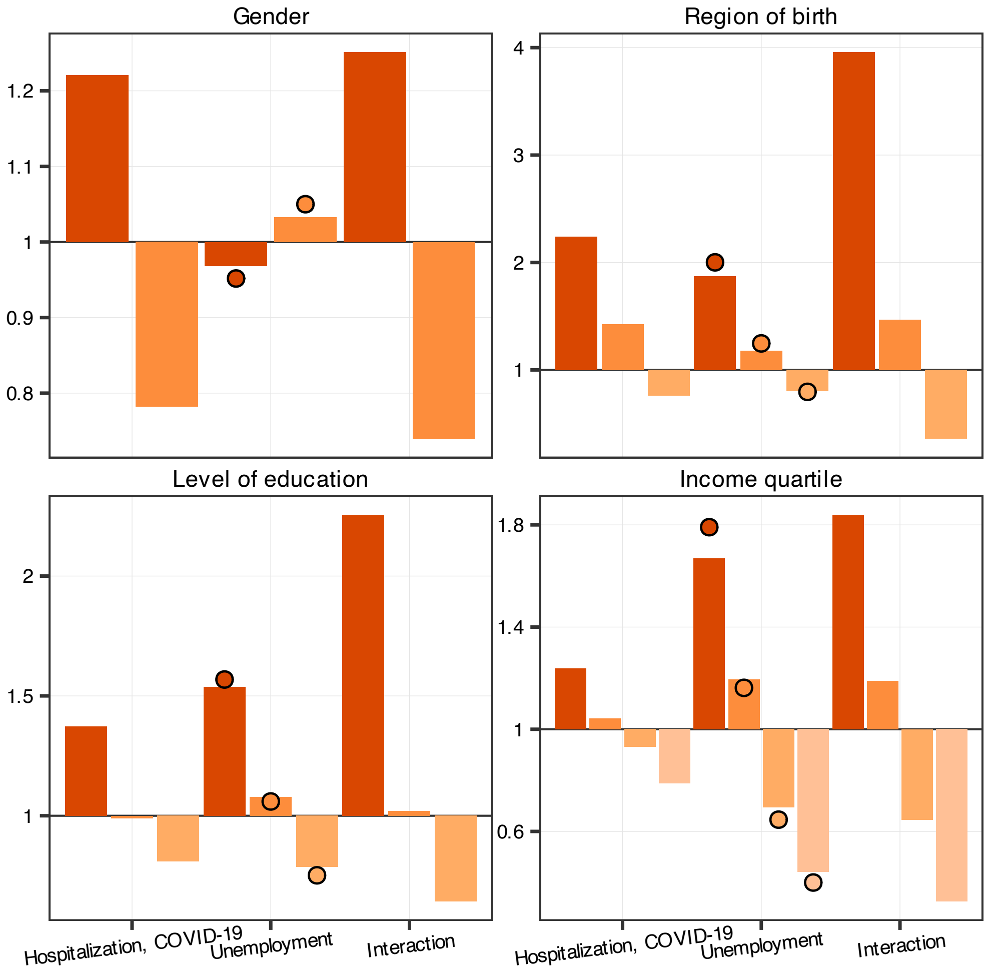

Notes: The two left most bar groups show the same relative risks as in the radar charts. The third group shows the relative risk for suffering from both a COVID-19 hospitalization and unemployment. Colored points show the 2016–2019 relative unemployment risk.

Figure S5. Individual interaction: Hospitalization and income loss

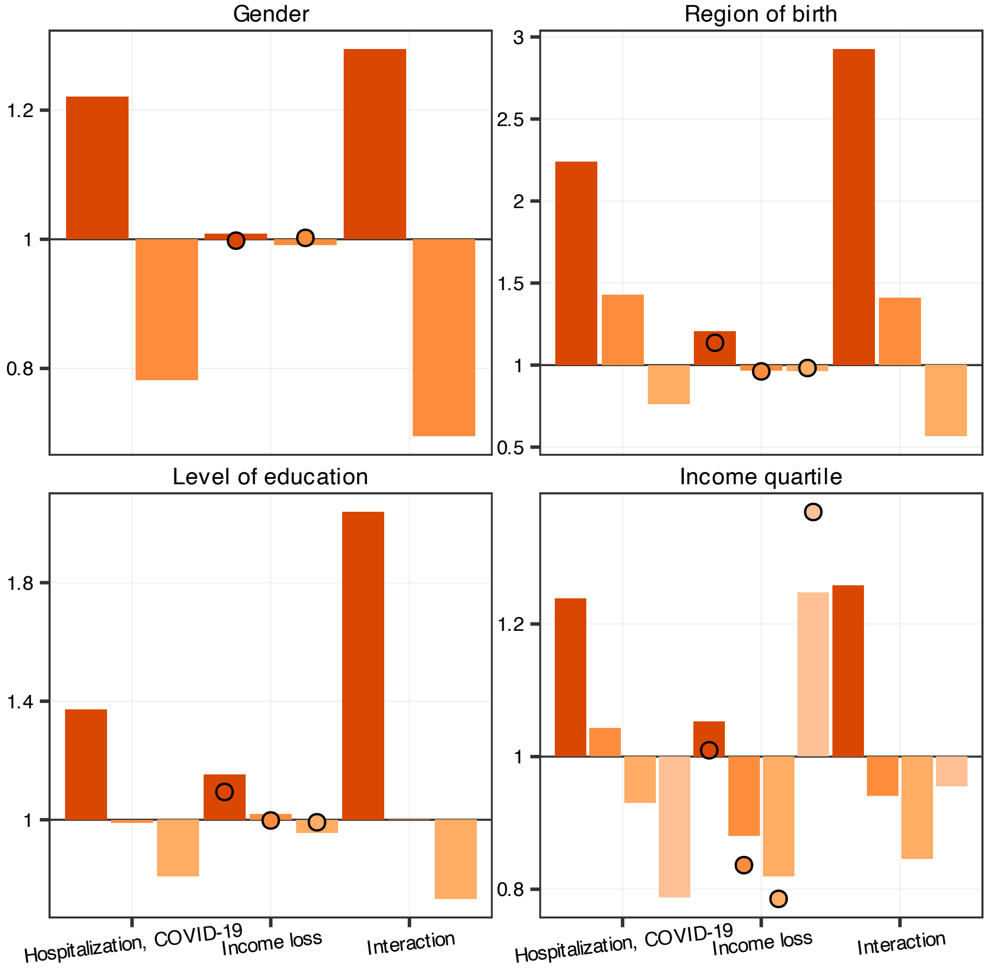

Notes: The two left most bar groups show the same relative risks as in the radar charts. The third group shows the relative risk for suffering from both a COVID-19 hospitalization and unemployment. Colored points show the 2016–2019 relative income loss risk.

D Supporting Tables

Table S1. Category frequencies (2019)

Gender		Men	Women		
		3 648 504	3 596 585		
		50.4%	49.6%		
Region of birth	Missing	Sweden	Europe	Outside of Europe	
	973	5 694 443	761 263	788 410	
	0.0%	78.6%	10.5%	10.9%	
Level of education	Missing	Compulsory	Upper secondary	Post-secondary	
	144 745	1 231 231	3 076 877	2 792 236	
	2.0%	17.0%	42.5%	38.5%	
Income quartile		Quartile 1	Quartile 2	Quartile 3	Quartile 4
		1 812 746	1 811 621	1 810 408	1 810 314
		25.0%	25.0%	25.0%	25.0%

Notes: The table shows the absolute number of individuals in the 2019 population, as well as the share, of the full 2019 population belonging to each of the group categories used in the analysis.

Table S2. Probability of cancer death and cancer diagnosis by population group, 2020 and 2016-2019

	Cancer death			Cancer diagnosis		
	2016-2019	2020	2021	2016-2019	2020	2021
**Gender**						
Men	0.36%	0.35%	0.34%	1.27%	1.20%	1.26%
Women	0.27%	0.26%	0.26%	1.30%	1.29%	1.36%
**Region of birth**						
Sweden	0.32%	0.31%	0.30%	1.34%	1.31%	1.38%
Europe	0.33%	0.32%	0.32%	1.09%	1.05%	1.10%
Outside of Europe	0.30%	0.28%	0.28%	1.02%	0.92%	1.01%
**Level of education**						
Compulsory	0.38%	0.40%	0.38%	1.19%	1.14%	1.19%
Upper secondary	0.32%	0.31%	0.30%	1.30%	1.25%	1.30%
Post-secondary	0.28%	0.26%	0.26%	1.34%	1.31%	1.39%
**Disposable income quartile**						
Quartile 1	0.34%	0.33%	0.32%	1.13%	1.07%	1.14%
Quartile 2	0.34%	0.32%	0.31%	1.27%	1.23%	1.28%
Quartile 3	0.30%	0.30%	0.29%	1.34%	1.29%	1.36%
Quartile 4	0.28%	0.27%	0.26%	1.40%	1.39%	1.46%

Notes: Cancer death is identified from the cause of death register, defined as all deaths where cancer (ICD C00-C96) was the underlying cause of death. Cancer diagnosis is identified from the cancer register. The reported estimates are predictive margins, based on a linear regression model adjusted for age (interacted with year of measurement) and region of residence.

Table S3. The probability of having ordered at least one PCR test.

	Probability
**Gender**	
Men	19.75%
Women	25.37%
**Region of birth**	
Sweden	25.35%
Europe	16.27%
Outside of Europe	11.10%
**Level of education**	
Compulsory	16.81%
Upper secondary	21.84%
Post-secondary	26.75%
**Disposable income quartile**	
Quartile 1	14.32%
Quartile 2	23.43%
Quartile 3	25.69%
Quartile 4	26.87%

Notes: The data was collected from the local administrative system. Only regions that use the 1177 system and consented to the use of data are included. These comprise 13 out of 21 county councils (Stockholm, Södermanland, Kalmar, Gotland, Blekinge, Skåne, Värmland, Örebro, Västmanland, Dalarna, Västernorrland, Jämtland and Norrbotten). The reported estimates are predictive margins, adjusted for age and region of residence.

D.1 Tables of absolute and relative effects

Table S4. Gender

	Men			Women		
	2016-2019	2020	2021	2016-2019	2020	2021
Positive, COVID-19		4.54%			5.26%	
		[0.93]			[1.07]	
Hospitalization, COVID-19		0.52%	0.50%		0.33%	0.31%
		[1.22]	[1.23]		[0.78]	[0.77]
Death, COVID-19		0.16%	0.09%		0.10%	0.05%
		[1.26]	[1.32]		[0.74]	[0.68]
Death, all causes	1.46%	1.55%	1.44%	1.08%	1.11%	1.04%
	[1.15]	[1.17]	[1.16]	[0.85]	[0.84]	[0.84]
Psychiatric care visit	3.19%	3.09%	3.15%	3.70%	3.60%	3.68%
	[0.92]	[0.92]	[0.92]	[1.07]	[1.08]	[1.08]
30d perioperative non-survival	1.46%	1.50%	1.50%	1.01%	1.04%	1.04%
	[1.21]	[1.21]	[1.22]	[0.83]	[0.84]	[0.84]
1yr cancer non-survival	8.63%	8.40%	7.61%	8.29%	8.01%	7.13%
	[1.02]	[1.03]	[1.04]	[0.98]	[0.98]	[0.97]
Income loss	19.31%	20.69%	16.70%	19.40%	20.32%	17.37%
	[1.00]	[1.01]	[0.98]	[1.00]	[0.99]	[1.02]
Unemployment	4.31%	6.43%	4.50%	4.75%	6.85%	4.98%
	[0.95]	[0.97]	[0.95]	[1.05]	[1.03]	[1.05]

Notes: The table shows the absolute and relative risks for men and women and for each of the nine outcomes for the 2016-2019 average and for 2020. The estimates are average predictive margins calculated from the regression estimates presented in columns 1-4 of Tables S8-S16. The relative risks, in brackets, are the ones presented in the figures of the main text. These are simply the absolute risks above divided by the corresponding predictive margin for the full population of the given period.

Table S5. Region of birth

	Outside of Europe			Europe			Sweden		
	2016-2019	2020	2021	2016-2019	2020	2021	2016-2019	2020	2021
Positive, COVID-19		6.10%			4.79%			4.75%	
		[1.24]			[0.98]			[0.97]	
Hospitalization, COVID-19		0.95%	0.80%		0.61%	0.59%		0.32%	0.32%
		[2.24]	[1.99]		[1.43]	[1.46]		[0.76]	[0.79]
Death, COVID-19		0.17%	0.09%		0.17%	0.09%		0.12%	0.07%
		[1.31]	[1.30]		[1.35]	[1.26]		[0.91]	[0.92]
Death, all causes	1.20%	1.31%	1.21%	1.33%	1.44%	1.35%	1.27%	1.31%	1.23%
	[0.95]	[0.99]	[0.98]	[1.05]	[1.08]	[1.09]	[1.00]	[0.99]	[0.99]
Psychiatric care visit	2.24%	1.79%	1.85%	2.86%	2.53%	2.54%	3.68%	3.68%	3.77%
	[0.65]	[0.54]	[0.54]	[0.83]	[0.76]	[0.74]	[1.07]	[1.10]	[1.10]
30d perioperative non-survival	1.19%	1.25%	1.23%	1.33%	1.33%	1.44%	1.19%	1.23%	1.22%
	[0.99]	[1.01]	[0.99]	[1.11]	[1.07]	[1.16]	[0.99]	[0.99]	[0.98]
1yr cancer non-survival	10.14%	9.80%	8.94%	10.60%	10.33%	9.18%	8.16%	7.89%	7.09%
	[1.20]	[1.20]	[1.22]	[1.26]	[1.26]	[1.25]	[0.97]	[0.96]	[0.96]
Income loss	21.98%	24.73%	20.77%	18.61%	19.85%	17.13%	19.01%	19.77%	16.26%
	[1.14]	[1.21]	[1.22]	[0.96]	[0.97]	[1.01]	[0.98]	[0.96]	[0.95]
Unemployment	9.06%	12.45%	9.05%	5.64%	7.83%	5.53%	3.60%	5.34%	3.76%
	[2.00]	[1.88]	[1.91]	[1.25]	[1.18]	[1.17]	[0.80]	[0.80]	[0.79]

Notes: The table shows the absolute and relative risks by region of birth and for each of the nine outcomes for the 2016-2019 average and for 2020. The estimates are average predictive margins calculated from the regression estimates presented in columns 1-4 of Tables S8-S16. The relative risks, in brackets, are the ones presented in the figures of the main text. These are simply the absolute risks above divided by the corresponding predictive margin for the full population of the given period.

Table S6. Level of education

	Compulsory			Upper secondary			Post-secondary		
	2016-2019	2020	2021	2016-2019	2020	2021	2016-2019	2020	2021
Positive, COVID-19		4.58%			4.94%			5.11%	
		[0.93]			[1.01]			[1.04]	
Hospitalization, COVID-19		0.58%	0.58%		0.42%	0.42%		0.34%	0.30%
		[1.37]	[1.45]		[0.99]	[1.03]		[0.81]	[0.76]
Death, COVID-19		0.15%	0.11%		0.13%	0.07%		0.11%	0.05%
		[1.18]	[1.47]		[1.02]	[1.04]		[0.86]	[0.73]
Death, all causes	1.68%	1.79%	1.72%	1.27%	1.33%	1.24%	1.07%	1.12%	1.04%
	[1.32]	[1.35]	[1.39]	[1.00]	[1.00]	[1.00]	[0.85]	[0.84]	[0.84]
Psychiatric care visit	5.25%	5.18%	5.31%	3.78%	3.75%	3.85%	2.30%	2.24%	2.30%
	[1.52]	[1.55]	[1.55]	[1.10]	[1.12]	[1.13]	[0.67]	[0.67]	[0.67]
30d perioperative non-survival	1.63%	1.72%	1.75%	1.20%	1.25%	1.23%	0.93%	0.96%	0.98%
	[1.35]	[1.39]	[1.42]	[1.00]	[1.01]	[0.99]	[0.78]	[0.77]	[0.79]
1yr cancer non-survival	10.99%	10.79%	10.02%	8.59%	8.43%	7.56%	6.14%	6.08%	5.32%
	[1.30]	[1.32]	[1.36]	[1.02]	[1.03]	[1.03]	[0.73]	[0.74]	[0.72]
Income loss	21.17%	23.63%	20.43%	19.31%	20.90%	17.08%	19.19%	19.61%	16.27%
	[1.09]	[1.15]	[1.20]	[1.00]	[1.02]	[1.00]	[0.99]	[0.96]	[0.96]
Unemployment	7.10%	10.20%	7.90%	4.80%	7.16%	5.10%	3.40%	5.22%	3.61%
	[1.57]	[1.54]	[1.67]	[1.06]	[1.08]	[1.08]	[0.75]	[0.79]	[0.76]

Notes: The table shows the absolute and relative risks by education and for each of the nine outcomes for the 2016-2019 average and for 2020. The estimates are average predictive margins calculated from the regression estimates presented in columns 1-4 of Tables S8-S16. The relative risks, in brackets, are the ones presented in the figures of the main text. These are simply the absolute risks above divided by the corresponding predictive margin for the full population of the given period.

Table S7. Income quartile

	Quartile 1			Quartile 2			Quartile 3			Quartile 4		
	2016-2019	2020	2021	2016-2019	2020	2021	2016-2019	2020	2021	2016-2019	2020	2021
Positive, COVID-19		3.70%			5.38%			5.40%			5.14%	
		[0.76]			[1.10]			[1.10]			[1.05]	
Hospitalization, COVID-19		0.53%	0.52%		0.44%	0.42%		0.40%	0.37%		0.34%	0.30%
		[1.24]	[1.29]		[1.04]	[1.05]		[0.93]	[0.92]		[0.79]	[0.74]
Death, COVID-19		0.13%	0.08%		0.14%	0.08%		0.13%	0.07%		0.11%	0.05%
		[0.99]	[1.16]		[1.10]	[1.14]		[1.02]	[1.02]		[0.89]	[0.68]
Death, all causes	1.42%	1.50%	1.41%	1.40%	1.46%	1.35%	1.20%	1.25%	1.17%	1.04%	1.10%	1.01%
	[1.12]	[1.13]	[1.14]	[1.11]	[1.10]	[1.09]	[0.95]	[0.95]	[0.95]	[0.82]	[0.83]	[0.81]
Psychiatric care visit	7.13%	6.98%	7.14%	3.48%	3.34%	3.38%	1.95%	1.90%	1.94%	1.23%	1.15%	1.19%
	[2.07]	[2.09]	[2.09]	[1.01]	[1.00]	[0.99]	[0.57]	[0.57]	[0.57]	[0.36]	[0.34]	[0.35]
30d perioperative non-survival	1.45%	1.53%	1.50%	1.32%	1.37%	1.33%	1.13%	1.15%	1.20%	0.93%	0.94%	0.95%
	[1.21]	[1.24]	[1.21]	[1.10]	[1.10]	[1.08]	[0.94]	[0.93]	[0.97]	[0.77]	[0.76]	[0.77]
1yr cancer non-survival	10.79%	11.03%	9.20%	9.61%	9.18%	8.77%	7.98%	7.65%	7.00%	5.99%	5.63%	5.01%
	[1.28]	[1.35]	[1.25]	[1.14]	[1.12]	[1.19]	[0.94]	[0.93]	[0.95]	[0.71]	[0.69]	[0.68]
Income loss	19.53%	21.60%	19.63%	16.18%	18.05%	14.32%	15.20%	16.80%	13.05%	26.49%	25.58%	21.15%
	[1.01]	[1.05]	[1.15]	[0.84]	[0.88]	[0.84]	[0.79]	[0.82]	[0.77]	[1.37]	[1.25]	[1.24]
Unemployment	8.11%	11.07%	8.76%	5.26%	7.93%	5.36%	2.93%	4.61%	2.98%	1.81%	2.93%	1.82%
	[1.79]	[1.67]	[1.85]	[1.16]	[1.20]	[1.13]	[0.65]	[0.69]	[0.63]	[0.40]	[0.44]	[0.38]

Notes: The table shows the absolute and relative risks by income quartile and for each of the nine outcomes for the 2016-2019 average and for 2020. The estimates are average predictive margins calculated from the regression estimates presented in columns 1-4 of Tables S8-S16. The relative risks, in brackets, are the ones presented in the figures of the main text. These are simply the absolute risks above divided by the corresponding predictive margin for the full population of the given period.

D.2 Tables of regression results

Table S8. Positive, COVID-19

	(1)	(2)	(3)	(4)	(5)
Quartile 2	1.67***				1.84***
	(0.02)				(0.02)
Quartile 3	1.69***				2.08***
	(0.02)				(0.02)
Quartile 4	1.44***				2.00***
	(0.02)				(0.02)
Upper secondary		0.35***			0.27***
		(0.02)			(0.02)
Post-secondary school		0.53***			0.17***
		(0.02)			(0.02)
Women			0.73***		0.92***
			(0.02)		(0.02)
Europe				0.04†	0.59***
				(0.03)	(0.03)
Outside of Europe				1.35***	2.15***
				(0.03)	(0.03)
Constant	1.96***	2.94***	2.75***	3.12***	0.94***
	(0.04)	(0.04)	(0.03)	(0.03)	(0.04)
Adjusted R2	0.01	0.01	0.01	0.01	0.01
Observations	7 327 948	7 327 948	7 327 948	7 327 776	7 327 776

Notes: Positive, COVID-19 (sample includes all Swedes age 25 and up). Coefficients and standard errors are reported in percentage points. The sample of analysis includes individual-level observations for each year 2016–2020. Base levels (income quartile 1, compulsory school, men, born in Sweden) are not reported. Missing values, coded as distinct categories, as well as region and age category fixed effects, are also excluded from the table. Standard errors are clustered at the individual level.

* $p \leq 0.05$, ** $p \leq 0.01$, *** $p \leq 0.001$.

Table S9. Hospitalization, COVID-19

	(1)	(2)	(3)	(4)	(5)
Quartile 2	−0.10***				−0.03***
	(0.01)				(0.01)
×2020	0.02†				0.04***
	(0.01)				(0.01)
×2021	0.00				0.00
	(0.00)				(0.00)
Quartile 3	−0.15***				−0.07***
	(0.01)				(0.01)
×2020	0.02†				0.05***
	(0.01)				(0.01)
×2021	0.00				0.00
	(0.00)				(0.00)
Quartile 4	−0.22***				−0.14***
	(0.01)				(0.01)
×2020	0.03***				0.06***
	(0.01)				(0.01)
×2021	0.00				0.00
	(0.00)				(0.00)
Upper secondary		−0.17***			−0.09***
		(0.01)			(0.01)
×2020		0.00			0.01
		(0.01)			(0.01)
×2021		0.00			0.00
		(0.00)			(0.00)
Post-secondary school		−0.28***			−0.17***
		(0.01)			(0.01)
×2020		0.04***			0.04**
		(0.01)			(0.01)
×2021		0.00			0.00
		(0.00)			(0.00)
Women			−0.18***		−0.20***
			(0.00)		(0.00)
×2020			0.00		0.00
			(0.01)		(0.01)
×2021			0.00		0.00
			(0.00)		(0.00)
Europe				0.27***	0.25***
				(0.01)	(0.01)
×2020				0.01	0.01
				(0.01)	(0.01)
×2021				0.00	0.00
				(0.00)	(0.00)
Outside of Europe				0.48***	0.43***
				(0.01)	(0.01)
×2020				0.15***	0.16***
				(0.01)	(0.01)
×2021				0.00	0.00
				(0.00)	(0.00)
2020	0.42***	0.42***	0.44***	0.43***	0.38***
	(0.03)	(0.03)	(0.03)	(0.03)	(0.03)
2021	0.00	0.00	0.00	0.00	0.00
	(0.00)	(0.00)	(0.00)	(0.00)	(0.00)
Constant	0.96***	0.97***	0.95***	0.80***	1.05***
	(0.02)	(0.02)	(0.02)	(0.02)	(0.02)
Adjusted R2	0.00	0.00	0.00	0.00	0.00
Observations	14 705 241	14 705 241	14 705 241	14 704 905	14 704 905

Notes: Hospitalization, COVID-19 (sample includes all Swedes age 25 and up). Coefficients and standard errors are reported in percentage points. The sample of analysis includes individual-level observations for each year 2016–2020. Base levels (income quartile 1, compulsory school, men, born in Sweden) are not reported. Missing values, coded as distinct categories, as well as region and age category fixed effects, are also excluded from the table. Standard errors are clustered at the individual level.

* $p \leq 0.05$, ** $p \leq 0.01$, *** $p \leq 0.001$.

Table S10. Death, COVID-19

	(1)	(2)	(3)	(4)	(5)
Quartile 2	0.00				0.00
	(0.00)				(0.00)
×2020	0.02***				0.02***
	(0.00)				(0.00)
×2021	0.00				0.00
	(0.00)				(0.00)
Quartile 3	−0.01***				−0.01*
	(0.00)				(0.00)
×2020	0.01**				0.02***
	(0.00)				(0.00)
×2021	0.00				0.00
	(0.00)				(0.00)
Quartile 4	−0.03***				−0.03***
	(0.00)				(0.00)
×2020	0.02***				0.03***
	(0.00)				(0.00)
×2021	0.00				0.00
	(0.00)				(0.00)
Upper secondary		−0.03***			−0.02***
		(0.00)			(0.00)
×2020		0.01			0.01
		(0.01)			(0.01)
×2021		0.00			0.00
		(0.00)			(0.00)
Post-secondary school		−0.05***			−0.04***
		(0.00)			(0.00)
×2020		0.01†			0.01
		(0.01)			(0.01)
×2021		0.00			0.00
		(0.00)			(0.00)
Women			−0.05***		−0.05***
			(0.00)		(0.00)
×2020			−0.02***		−0.02***
			(0.00)		(0.00)
×2021			0.00		0.00
			(0.00)		(0.00)
Europe				0.02***	0.02***
				(0.00)	(0.00)
×2020				0.03***	0.03***
				(0.01)	(0.01)
×2021				0.00	0.00
				(0.00)	(0.00)
Outside of Europe				0.03***	0.02***
				(0.00)	(0.00)
×2020				0.02***	0.03***
				(0.00)	(0.00)
×2021				0.00	0.00
				(0.00)	(0.00)
2020	0.34***	0.34***	0.36***	0.35***	0.33***
	(0.02)	(0.02)	(0.02)	(0.02)	(0.02)
2021	0.00	0.00	0.00	0.00	0.00
	(0.00)	(0.00)	(0.00)	(0.00)	(0.00)
Constant	0.35***	0.36***	0.37***	0.34***	0.39***
	(0.01)	(0.01)	(0.01)	(0.01)	(0.01)
Adjusted R2	0.01	0.01	0.01	0.01	0.01
Observations	14 705 241	14 705 241	14 705 241	14 704 905	14 704 905

Notes: Death, COVID-19 (sample includes all Swedes age 25 and up). Coefficients and standard errors are reported in percentage points. The sample of analysis includes individual-level observations for each year 2016–2020. Base levels (income quartile 1, compulsory school, men, born in Sweden) are not reported. Missing values, coded as distinct categories, as well as region and age category fixed effects, are also excluded from the table. Standard errors are clustered at the individual level.

$^{*}\ p \leq 0.05$, $^{**}\ p \leq 0.01$, $^{***}\ p \leq 0.001$.

Table S11. Death, all causes

	(1)	(2)	(3)	(4)	(5)
Quartile 2	−0.02***				−0.02*
	(0.01)				(0.01)
×2020	−0.02				−0.01
	(0.01)				(0.01)
×2021	−0.03**				−0.03*
	(0.01)				(0.01)
Quartile 3	−0.22***				−0.26***
	(0.01)				(0.01)
×2020	−0.02				−0.01
	(0.01)				(0.01)
×2021	−0.01				0.00
	(0.01)				(0.01)
Quartile 4	−0.38***				−0.45***
	(0.01)				(0.01)
×2020	−0.02				0.00
	(0.01)				(0.01)
×2021	−0.02†				0.00
	(0.01)				(0.01)
Upper secondary		−0.41***			−0.37***
		(0.01)			(0.01)
×2020		−0.04*			−0.04†
		(0.02)			(0.02)
×2021		−0.06***			−0.06***
		(0.02)			(0.02)
Post-secondary school		−0.61***			−0.45***
		(0.01)			(0.01)
×2020		−0.06***			−0.05**
		(0.02)			(0.02)
×2021		−0.08***			−0.07***
		(0.02)			(0.02)
Women			−0.39***		−0.45***
			(0.00)		(0.00)
×2020			−0.05***		−0.05***
			(0.01)		(0.01)
×2021			−0.02†		−0.01
			(0.01)		(0.01)
Europe				0.06***	−0.02**
				(0.01)	(0.01)
×2020				0.06***	0.05***
				(0.02)	(0.02)
×2021				0.06***	0.06***
				(0.01)	(0.02)
Outside of Europe				−0.07***	−0.25***
				(0.00)	(0.00)
×2020				0.06***	0.05***
				(0.01)	(0.01)
×2021				0.05***	0.05***
				(0.01)	(0.01)
2020	0.28***	0.32***	0.29***	0.26***	0.33***
	(0.05)	(0.05)	(0.05)	(0.05)	(0.05)
2021	−0.30***	−0.24***	−0.31***	−0.32***	−0.26***
	(0.05)	(0.05)	(0.05)	(0.05)	(0.05)
Constant	5.90***	6.01***	5.96***	5.74***	6.42***
	(0.02)	(0.02)	(0.02)	(0.02)	(0.02)
Adjusted R2	0.08	0.08	0.08	0.08	0.08
Observations	43 108 728	43 108 728	43 108 728	43 107 760	43 107 756

Notes: Death, all causes (sample includes all Swedes age 25 and up). Coefficients and standard errors are reported in percentage points. The sample of analysis includes individual-level observations for each year 2016–2020. Base levels (income quartile 1, compulsory school, men, born in Sweden) are not reported. Missing values, coded as distinct categories, as well as region and age category fixed effects, are also excluded from the table. Standard errors are clustered at the individual level.

* $p \le 0.05$, ** $p \le 0.01$, *** $p \le 0.001$.

Table S12. Psychiatric care visit

	(1)	(2)	(3)	(4)	(5)
Quartile 2	−3.65***				−4.16***
	(0.02)				(0.02)
×2020	0.01				−0.06*
	(0.02)				(0.02)
×2021	−0.12***				−0.16***
	(0.02)				(0.02)
Quartile 3	−5.18***				−5.81***
	(0.02)				(0.02)
×2020	0.10***				0.00
	(0.02)				(0.02)
×2021	−0.02				−0.08***
	(0.02)				(0.02)
Quartile 4	−5.90***				−6.51***
	(0.02)				(0.02)
×2020	0.07***				−0.05*
	(0.02)				(0.02)
×2021	−0.05**				−0.11***
	(0.02)				(0.02)
Upper secondary		−1.48***			−0.91***
		(0.02)			(0.02)
×2020		0.05**			−0.02
		(0.02)			(0.02)
×2021		0.01			−0.08***
		(0.02)			(0.02)
Post-secondary school		−2.96***			−1.64***
		(0.02)			(0.02)
×2020		0.02			−0.03
		(0.02)			(0.02)
×2021		−0.05**			−0.09***
		(0.02)			(0.02)
Women			0.52***		−0.23***
			(0.01)		(0.01)
×2020			0.00		0.01
			(0.01)		(0.01)
×2021			0.02		0.03**
			(0.01)		(0.01)
Europe				−0.81***	−1.87***
				(0.02)	(0.02)
×2020				−0.34***	−0.28***
				(0.02)	(0.02)
×2021				−0.42***	−0.25***
				(0.02)	(0.02)
Outside of Europe				−1.44***	−3.53***
				(0.02)	(0.02)
×2020				−0.45***	−0.38***
				(0.02)	(0.02)
×2021				−0.48***	−0.42***
				(0.02)	(0.02)
2020	−0.45***	−0.33***	−0.40***	−0.35***	−0.27***
	(0.03)	(0.02)	(0.02)	(0.02)	(0.03)
2021	−0.30***	−0.20***	−0.35***	−0.28***	−0.11***
	(0.03)	(0.03)	(0.02)	(0.02)	(0.03)
Constant	5.26***	2.72***	1.26***	1.68***	6.84***
	(0.02)	(0.02)	(0.02)	(0.02)	(0.03)
Adjusted R2	0.02	0.01	0.01	0.01	0.03
Observations	43 108 728	43 108 728	43 108 728	43 107 760	43 107 756

Notes: Specialized psychiatric outpatient care visits (sample includes all Swedes age 25 and up). Coefficients and standard errors are reported in percentage points. The sample of analysis includes individual-level observations for each year 2016–2020. Base levels (income quartile 1, compulsory school, men, born in Sweden) are not reported. Missing values, coded as distinct categories, as well as region and age category fixed effects, are also excluded from the table. Standard errors are clustered at the individual level.

* $p \leq 0.05$, ** $p \leq 0.01$, *** $p \leq 0.001$.

Table S13. 30-day perioperative non-survival

	(1)	(2)	(3)	(4)	(5)
Quartile 2	−0.13***				−0.12***
	(0.01)				(0.01)
×2020	−0.04				−0.03
	(0.03)				(0.03)
×2021	−0.03				−0.03
	(0.03)				(0.03)
Quartile 3	−0.33***				−0.36***
	(0.01)				(0.01)
×2020	−0.05†				−0.05
	(0.03)				(0.03)
×2021	0.03				0.04
	(0.03)				(0.03)
Quartile 4	−0.52***				−0.58***
	(0.01)				(0.02)
×2020	−0.07*				−0.06†
	(0.03)				(0.03)
×2021	−0.03				−0.01
	(0.03)				(0.03)
Upper secondary		−0.43***			−0.36***
		(0.02)			(0.02)
×2020		−0.04			−0.03
		(0.04)			(0.04)
×2021		−0.10**			−0.10**
		(0.04)			(0.04)
Post-secondary school		−0.69***			−0.49***
		(0.02)			(0.02)
×2020		−0.07†			−0.05
		(0.04)			(0.04)
×2021		−0.09*			−0.09*
		(0.04)			(0.04)
Women			−0.46***		−0.54***
			(0.01)		(0.01)
×2020			0.00		0.00
			(0.02)		(0.02)
×2021			−0.01		0.00
			(0.02)		(0.02)
Europe				0.14***	0.03*
				(0.02)	(0.02)
×2020				−0.04	−0.06
				(0.04)	(0.04)
×2021				0.08*	0.08*
				(0.04)	(0.04)
Outside of Europe				0.00	−0.24***
				(0.01)	(0.01)
×2020				0.02	−0.01
				(0.03)	(0.03)
×2021				0.01	0.00
				(0.02)	(0.03)
2020	0.14*	0.15*	0.09	0.10	0.18*
	(0.07)	(0.07)	(0.07)	(0.07)	(0.08)
2021	0.02	0.11	0.01	0.00	0.08
	(0.07)	(0.07)	(0.06)	(0.06)	(0.07)
Constant	3.28***	3.31***	3.25***	2.99***	3.83***
	(0.03)	(0.03)	(0.03)	(0.03)	(0.03)
Adjusted R2	0.02	0.02	0.02	0.02	0.02
Observations	7 622 792	7 622 792	7 622 792	7 622 701	7 622 701

Notes: Death within 30 days of surgical procedure (sample includes all Swedes age 25 and up who went through surgery in the given year). Coefficients and standard errors are reported in percentage points. The sample of analysis includes individual-level observations for each year 2016–2020. Base levels (income quartile 1, compulsory school, men, born in Sweden) are not reported. Missing values, coded as distinct categories, as well as region and age category fixed effects, are also excluded from the table. Standard errors are clustered at the individual level.

* $p \leq 0.05$, ** $p \leq 0.01$, *** $p \leq 0.001$.

Table S14. 1-year cancer non-survival

	(1)	(2)	(3)	(4)	(5)
Quartile 2	−1.18***				−0.96***
	(0.15)				(0.15)
×2020	−0.66*				−0.70*
	(0.33)				(0.33)
×2021	0.75*				0.72*
	(0.31)				(0.31)
Quartile 3	−2.81***				−2.38***
	(0.14)				(0.15)
×2020	−0.57†				−0.67*
	(0.32)				(0.33)
×2021	0.60*				0.55†
	(0.29)				(0.31)
Quartile 4	−4.80***				−3.96***
	(0.14)				(0.15)
×2020	−0.60*				−0.79*
	(0.30)				(0.33)
×2021	0.61*				0.45
	(0.28)				(0.31)
Upper secondary		−2.40***			−1.93***
		(0.14)			(0.14)
×2020		0.04			0.09
		(0.31)			(0.32)
×2021		−0.06			−0.13
		(0.30)			(0.30)
Post-secondary school		−4.85***			−3.58***
		(0.14)			(0.14)
×2020		0.14			0.33
		(0.31)			(0.32)
×2021		0.15			0.08
		(0.29)			(0.30)
Women			−0.33**		−1.10***
			(0.10)		(0.11)
×2020			−0.05		−0.15
			(0.22)		(0.24)
×2021			−0.15		−0.08
			(0.21)		(0.22)
Europe				2.45***	1.67***
				(0.18)	(0.18)
×2020				0.00	−0.02
				(0.39)	(0.40)
×2021				−0.36	−0.23
				(0.37)	(0.38)
Outside of Europe				1.98***	0.52*
				(0.22)	(0.23)
×2020				−0.07	−0.34
				(0.47)	(0.47)
×2021				−0.13	−0.09
				(0.45)	(0.46)
2020	0.05	−0.30	−0.42	−0.45	0.25
	(0.45)	(0.44)	(0.41)	(0.39)	(0.51)
2021	−2.50***	−1.80***	−1.91***	−1.94***	−2.20***
	(0.41)	(0.41)	(0.38)	(0.36)	(0.47)
Constant	15.81***	15.23***	13.48***	13.07***	17.22***
	(0.21)	(0.20)	(0.19)	(0.18)	(0.23)
Adjusted R2	0.03	0.03	0.02	0.02	0.03
Observations	530 749	530 749	530 749	530 748	530 748

Notes: Cancer death within one year (sample includes all Swedes age 25 and up who were diagnosed with cancer in the given year). Coefficients and standard errors are reported in percentage points. The sample of analysis includes individual-level observations for each year 2016–2020. Base levels (income quartile 1, compulsory school, men, born in Sweden) are not reported. Missing values, coded as distinct categories, as well as region and age category fixed effects, are also excluded from the table. Standard errors are clustered at the individual level.

* $p \leq 0.05$, ** $p \leq 0.01$, *** $p \leq 0.001$.

Table S15. Income loss

	(1)	(2)	(3)	(4)	(5)
Quartile 2	−3.35***				−2.95***
	(0.03)				(0.03)
×2020	−0.20***				0.08
	(0.05)				(0.06)
×2021	−1.96***				−1.69***
	(0.05)				(0.05)
Quartile 3	−4.34***				−3.25***
	(0.03)				(0.03)
×2020	−0.46***				−0.09
	(0.05)				(0.06)
×2021	−2.25***				−1.90***
	(0.05)				(0.05)
Quartile 4	6.95***				8.74***
	(0.03)				(0.03)
×2020	−2.97***				−2.56***
	(0.06)				(0.06)
×2021	−5.43***				−5.08***
	(0.05)				(0.06)
Upper secondary		−1.86***			−1.63***
		(0.03)			(0.03)
×2020		−0.86***			−0.15*
		(0.07)			(0.07)
×2021		−1.49***			−0.56***
		(0.06)			(0.07)
Post-secondary school		−1.98***			−3.51***
		(0.03)			(0.03)
×2020		−2.04***			−0.90***
		(0.07)			(0.07)
×2021		−2.19***			−0.70***
		(0.06)			(0.07)
Women			0.08***		1.86***
			(0.02)		(0.02)
×2020			−0.45***		−0.83***
			(0.04)		(0.04)
×2021			0.59***		−0.10*
			(0.04)		(0.04)
Europe				−0.40***	0.96***
				(0.03)	(0.03)
×2020				0.48***	0.31***
				(0.06)	(0.06)
×2021				1.27***	0.35***
				(0.06)	(0.06)
Outside of Europe				2.98***	4.20***
				(0.03)	(0.03)
×2020				1.98***	1.53***
				(0.06)	(0.06)
×2021				1.53***	0.18**
				(0.06)	(0.06)
2020	2.10***	2.36***	1.41***	0.94***	2.50***
	(0.07)	(0.08)	(0.07)	(0.06)	(0.09)
2021	0.08	−0.82***	−2.63***	−2.67***	0.33***
	(0.07)	(0.08)	(0.06)	(0.06)	(0.09)
Constant	22.86***	24.35***	22.70***	22.58***	22.74***
	(0.03)	(0.04)	(0.03)	(0.03)	(0.04)
Adjusted R2	0.01	0.01	0.00	0.01	0.02
Observations	31 004 086	31 004 086	31 004 086	31 003 132	31 003 132

Notes: Income loss (sample includes all working-age Swedes age 25–64). Coefficients and standard errors are reported in percentage points. The sample of analysis includes individual-level observations for each year 2016–2020. Base levels (income quartile 1, compulsory school, men, born in Sweden) are not reported. Missing values, coded as distinct categories, as well as region and age category fixed effects, are also excluded from the table. Standard errors are clustered at the individual level.

* $p \leq 0.05$, ** $p \leq 0.01$, *** $p \leq 0.001$.

Table S16. Unemployment

	(1)	(2)	(3)	(4)	(5)
Quartile 2	−2.85***				−2.21***
	(0.02)				(0.02)
×2020	−0.29***				−0.23***
	(0.04)				(0.04)
×2021	−0.56***				−0.69***
	(0.03)				(0.04)
Quartile 3	−5.19***				−4.23***
	(0.02)				(0.02)
×2020	−1.28***				−1.10***
	(0.03)				(0.04)
×2021	−0.59***				−0.70***
	(0.03)				(0.03)
Quartile 4	−6.30***				−5.07***
	(0.01)				(0.02)
×2020	−1.84***				−1.60***
	(0.03)				(0.04)
×2021	−0.64***				−0.74***
	(0.03)				(0.03)
Upper secondary		−2.30***			−0.72***
		(0.02)			(0.02)
×2020		−0.74***			−0.02
		(0.04)			(0.04)
×2021		−0.50***			−0.21***
		(0.04)			(0.04)
Post-secondary school		−3.70***			−1.66***
		(0.02)			(0.02)
×2020		−1.29***			−0.33***
		(0.04)			(0.05)
×2021		−0.59***			−0.20***
		(0.04)			(0.04)
Women			0.45***		−0.05***
			(0.01)		(0.01)
×2020			−0.02		−0.24***
			(0.02)		(0.02)
×2021			0.03†		−0.01
			(0.02)		(0.02)
Europe				2.04***	1.04***
				(0.02)	(0.02)
×2020				0.45***	0.63***
				(0.04)	(0.04)
×2021				−0.26***	−0.02
				(0.03)	(0.04)
Outside of Europe				5.46***	3.75***
				(0.02)	(0.02)
×2020				1.65***	1.48***
				(0.04)	(0.04)
×2021				−0.17***	−0.20***
				(0.04)	(0.04)
2020	1.65***	1.69***	0.81***	0.56***	1.60***
	(0.03)	(0.04)	(0.03)	(0.02)	(0.05)
2021	0.53***	0.63***	0.07**	0.02	0.79***
	(0.03)	(0.04)	(0.02)	(0.02)	(0.05)
Constant	5.71***	4.44***	1.88***	1.50***	5.57***
	(0.02)	(0.02)	(0.01)	(0.01)	(0.02)
Adjusted R2	0.02	0.01	0.01	0.02	0.03
Observations	31 004 086	31 004 086	31 004 086	31 003 132	31 003 132

Notes: Unemployment (sample includes all working-age Swedes age 25–64). Coefficients and standard errors are reported in percentage points. The sample of analysis includes individual-level observations for each year 2016–2020. Base levels (income quartile 1, compulsory school, men, born in Sweden) are not reported. Missing values, coded as distinct categories, as well as region and age category fixed effects, are also excluded from the table. Standard errors are clustered at the individual level.

* $p \leq 0.05$, ** $p \leq 0.01$, *** $p \leq 0.001$.
